# Prediction of Protein Tertiary Structure via Regularized Template Classification Techniques

**DOI:** 10.3390/molecules25112467

**Published:** 2020-05-26

**Authors:** Óscar Álvarez-Machancoses, Juan Luis Fernández-Martínez, Andrzej Kloczkowski

**Affiliations:** 1 Group of Inverse Problems, Optimization and Machine Learning, Department of Mathematics, University of Oviedo, C. Federico García Lorca, 18, 33007 Oviedo, Spain; UO217123@uniovi.es (Ó.Á.-M.); jlfm@uniovi.es (J.L.F.-M.); 2 Battelle Center for Mathematical Medicine, Nationwide Children’s Hospital, Columbus, OH Department of Pediatrics, The Ohio State University, Columbus, OH 43210, USA

**Keywords:** Protein Tertiary Structure, LDA classification, PSO, uncertainty analysis

## Abstract

We discuss the use of the regularized linear discriminant analysis (LDA) as a model reduction technique combined with particle swarm optimization (PSO) in protein tertiary structure prediction, followed by structure refinement based on singular value decomposition (SVD) and PSO. The algorithm presented in this paper corresponds to the category of template-based modeling. The algorithm performs a preselection of protein templates before constructing a lower dimensional subspace via a regularized LDA. The protein coordinates in the reduced spaced are sampled using a highly explorative optimization algorithm, regressive–regressive PSO (RR-PSO). The obtained structure is then projected onto a reduced space via singular value decomposition and further optimized via RR-PSO to carry out a structure refinement. The final structures are similar to those predicted by best structure prediction tools, such as Rossetta and Zhang servers. The main advantage of our methodology is that alleviates the ill-posed character of protein structure prediction problems related to high dimensional optimization. It is also capable of sampling a wide range of conformational space due to the application of a regularized linear discriminant analysis, which allows us to expand the differences over a reduced basis set.

## 1. Introduction

Recent advances in genome sequencing techniques have dramatically increased the amount of available sequence information of proteins [1]. Over 150,000 protein structures are currently solved and deposited in the Protein Data Bank (PDB), with a yearly growth rate of 10%, while the number of known protein sequences in UniProt exceeds 158,000,000 [1]. Since experimental methods of protein structure determination such as X-ray crystallography, or nuclear magnetic resonance (NMR) are expensive and time consuming, there is an excellent opportunity to apply computational protein structure prediction methods to narrow the gap between the number of protein sequences and the number of structures [2].

Computational methods can be divided in two broad classes: (1) template-free modeling, which is based on predicting protein structure from physics first principles by global minimization of the free energy of a protein [2,3]; and (2) template-based methodologies, based on either threading or comparative modeling [4]. These methodologies are strongly based on sequence similarity between the sequence of the modeled protein and proteins with known structure from the PDB. A sequence identity is determined by using PSI-BLAST search to compare a query the sequence with a database of sequences with known structures (PDB). If the sequence identity of a query sequence is low (less than 15%) it indicates that new fold and template-free modelling methods have to be used. For high sequence identity homology, modeling methods are used.

Template-based comparative methods are referred to those where not only the fold is determined by the template, but also a full atom model is built [5]. In this sense, this modeling technique requires that at least one of the templates used in the modeling should be determined by experiment. The whole set of protein models utilized in the prediction can be generated based on structural alignment [6]. Because of this, it is possible to model the 3D structure of the native-like protein, and to include within the prediction outcome the small structural differences within a protein superfamily [7]. Nowadays, the probability of finding a related protein, whose structure is known, to a randomly selected one, ranges from 30% to 80%, depending on the genome. Furthermore, approximately 70% of all known protein sequences have at least one domain that is noticeably linked to a known protein structure [8].

Generally speaking, if similarity between two proteins is detected at the sequence level, the structural similarity is assumed. However, this approach does not take into account the small 3D structural differences that may exist within a given sequence [9]. Therefore, the use of computational methods and machine learning techniques are advantageous alternatives, since once the training models have been built from the “a priori” information, structure predictions can be performed quickly [9]. These training models generally set additional restrictions from general statistical mechanics force fields, which lead to the development of better sampling techniques that could explore the entire conformational space [10,11].

The growing importance of structural bioinformatics is documented by the existence of Structural Classification of Proteins (SCOP) and Class-Architecture-Topology-Homology superfamily (CATH) databases of folds of proteins [12,13,14], by the increasing availability of various web servers that automate the template-based modelling process [10,11,15,16,17], and also non-automated servers that generally offer better results [18].

Generally speaking, template-based modelling requires complex decisions such as optimally selecting templates, refining alignments, mechanistic force fields, and further restraints based on expert knowledge [19,20]. In this sense, several template-based modeling methods have been developed over the last years. Schaffer et al. [21] utilized composition-based statistics to classify protein templates prior optimizing the energy function of the target sequence. Brenner et al. [22] and Sauder et al. [23] assessed sequence similarity utilizing sampling and evolutionary methods. It is worth mentioning that the accuracy of template-based modelling increases when more than one template is utilized to construct a protein 3D structure, as reported by Venclovas et al. [24] and Sanchez et al. [25], and then each template is evaluated according to a scoring function such as the energy function [26]. The resulting model predictions outperform models that were based on the single best template [27]. When several templates are utilized to model the protein, they generally are superposed with each other and, later on, the multiple template-based alignment is utilized [28,29]. Methods such as the multiple mapping method (MMM) developed by Rai et al. [30] successfully models protein structures by minimizing the alignment errors and optimally merging differently aligned fragments from a database of different alignments or even based on higher order conditional random fields [31]. Once templates have been selected and constructed, model building can be carried out in several ways. In this sense, a protein 3D structure can be predicted using models based on the assembly of rigid bodies [32]. Another successful approach is modelling protein structures by using a set of atomic coordinates from templates, such as C-alphas, as guiding positions, to assemble the rest of the folds and atoms coordinates. Computational methods and machine learning have been widely utilized in protein model construction. Genetic algorithms [33] have been utilized to iteratively perform protein structure prediction, to carry out the template selection, alignment, model building, and model assessment at each iteration [34]. In addition, other approaches in model building include the use of molecular dynamics simulations [35], simulated annealing [36], evolutionary information [37], Monte Carlo [38], deep learning [39], perturbation methods [40], multiple-copy simultaneous search, or self-consistent field optimization [41].

In this research paper, we propose the utilization of a regularized linear discriminant analysis in order to classify a set of protein templates based on its dipolar Distance-scale Finite Ideal Gas Reference Equation (dDFIRE) energy score in combination with a particle swarm optimizer (PSO). PSO has been successfully utilized in the prediction of both secondary and tertiary protein structures, and it is a good alternative to reconstruct the protein model and sample the full conformational space of the protein family at the same time [9,42,43]. After this, an additional refinement step is performed utilizing a simple and fast SVD model reduction with a further PSO optimization.

## 2. Methods

**Figure 1:**
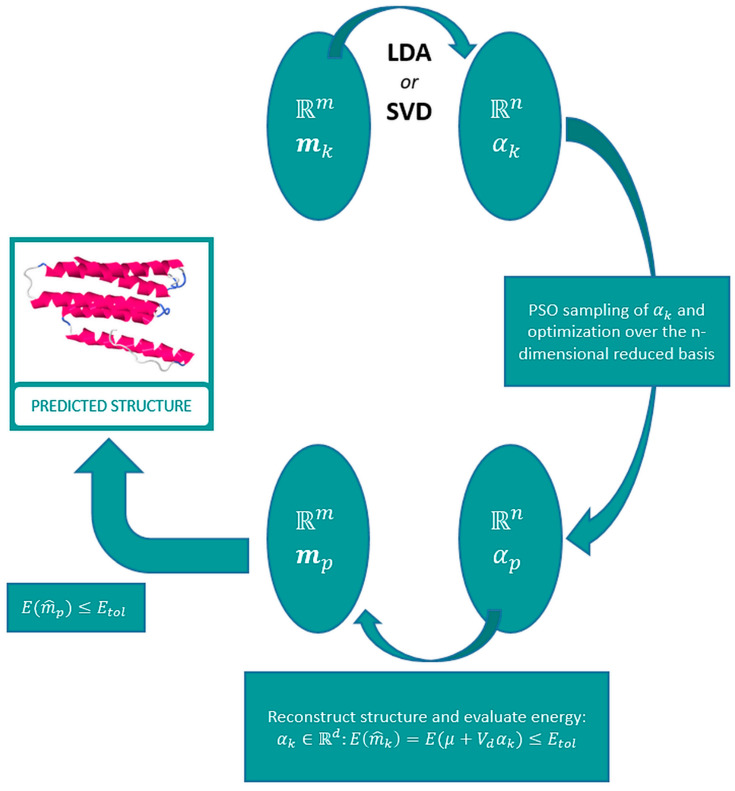
Linear discriminant analysis (LDA)–singular value decomposition (SVD) algorithm flowchart.

The algorithm proposed in this paper consists of 4 sequential steps: (1) template selection, (2) model reduction and alignment, (3) model optimization, (4) protein predicted structure refinement, and 5) evaluation of the final refined predicted model based on energy and structural considerations. Figure 1 shows the flowchart of the prediction algorithm using a reduced basis provided by a regularized LDA and SVD.

### 2.1. Template Selection and Model Reduction via an L_2_-Regularized LDA Discriminant Classifier

Linear discriminant analysis (LDA) is an algorithm broadly utilized in classification problems and model reduction techniques proposed first by Fisher [44]. Generally speaking, the LDA is utilized in this research in order to, initially, classify the protein templates according to its suitability for protein 3D structure determination, and as a model reduction technique. LDA provides the reduced basis set that maximizes the intra-class distance among different families of templates. In this sense, the protein templates are transformed into a low-dimensional subspace in such a way that the template class centroids are separated as much as possible. This technique has recently been used in the prediction of secondary structures [9] and also in phenotype prediction classification problems using genetic data [45,46,47].

Our approach firstly carries out energy and RMSD evaluations of the protein templates before partitioning the N-dimensional population into N sets, known as classes. The selection of proper templates is of utmost importance in order to correctly predict protein structure. To address this problem Kalina and Matonoha [48] proposed a centroid-based classification, which performs a supervised variable selection to optimize a prototype. Cernea et al. [49] proposed a similar sampling method in a phenotype prediction problem utilizing a Fisher’s ratio sampler. Therefore, an ensemble of *l* plausible protein templates of *n* atoms, $m_{i}\in {\mathbb{R}}^{n}$, is selected and arranged column wise into the decoy’s experimental matrix: $X=\left(m_{1},m_{2},\ldots ,m_{l}\right)\in M_{n\times l}$. Then, the BioShell package is utilized to compute the energy of each template utilizing a dDFIRE, which accurately represents the energy of the native structure, hydrogen bonding, hydrophobic interactions, and structural properties over a wide range of proteins [50]. In addition, an implicit solvation model of water is utilized, developed by Qiu and co-workers [51], known as generalized Born/surface area free-energy (GB/SA). Alongside with the energy considerations, the RMSD is calculated. A *k*-means energy partitioning is carried out in order to separate the protein templates in classes and select those classes that are more suitable for the protein prediction while expanding as much information about the conformational space.

Provided the classes, LDA considers a set of $n_{k}$ templates belonging to a class k; therefore, we denote by $\mu_{k}$ the mean of class $C_{k}$ and by μ the mean of all the samples, n.

(1) $$\mu_{k}=\frac{1}{n_{k}}\sum_{m_{i}\in C_{k}}m_{i}\;and\;\mu =\frac{1}{n}\sum_{i}m_{i}.$$

The proteins are represented by two matrices, $S_{B}$ and $S_{W}$, known as the between-class scatter matrix and the within-class scatter matrix, respectively, that is, the inter-class and intra-class covariance. Their definitions are as follows:

(2) $$S_{B}=\underset{k}{\sum }n_{k}\left(\mu_{k}-\mu \right){\left(\mu_{k}-\mu \right)}^{T}$$

(3) $$S_{W}=\underset{k}{\sum }\underset{m_{i}\in C_{k}}{\sum }\left(m_{i}-\mu_{k}\right){\left(m_{i}-\mu_{k}\right)}^{T}$$

LDA looks for a linear combination of the initial variables such that the means of the classes are well separated with respect to the summation of the variances of the data assigned to each class. For this purpose, LDA determines a vector w, so that $w^{t}S_{B}w$ is maximized and $w^{t}S_{W}w$ minimized. It can be proved that the solution to this problem is $w_{opt}$, which is the eigenvector associated with the eigenvalue of $S_{W}^{-1}S_{B}$, when $S_{W}^{-1}$ exists. However, since this problem is ill-posed due to the fact that the number of observations is much higher than the number of variables, a simple LDA is not robust enough and, depending on the templates, it may lead to instability due to a singular $S_{W}$. To avoid this instability, the L_2_-regularized LDA is used [52,53]. The scatter matrix $S_{d}$ is regularized as follows:

(4) $$S_{d}^{reg}=\left(1-\lambda_{d}\right)S_{d}+\lambda_{d}s_{d}I_{n}$$

where the subscript d refers to each scatter matrix, $S_{B}$ and $S_{w}$, $S_{d}^{reg}$ is the regularized scatter matrix, $\lambda_{d}$ is the regularization parameter, $s_{d}$ is the second regularization parameter, and $I_{n}$ is the identity matrix. The regularization parameters are

(5) $$\lambda_{d}=\frac{2\sum_{i=2}^{p}\sum_{j=1}^{i-1}var\left(S_{ij}\right)}{2\sum_{i=2}^{p}\sum_{j=1}^{i-1}S_{ij}{}^{2}+\sum_{i=1}^{p}{\left(S_{ii}-1\right)}^{2}}$$

(6) sd=∑i=1pSiip

where $var\left(S_{ij}\right)$ is the maximum likelihood estimator of the variance of $S_{ij}$.

Computing the regularized covariances and calculating $w_{opt}$ yields the reduced template landscape. By doing this, the ill-posed character is alleviated in a much lower dimensional space, finding

(7) $$a_{k}\in {\mathbb{R}}^{d}:E\left({\hat{m}}_{k}\right)=E\left(\mu +V_{d}a_{k}\right)\leq E_{tol}$$

where ${\hat{m}}_{k}$ is the predicted reconstructed protein structure given a certain tolerance, $\mu ,V_{d}$ are provided by the regularized linear discriminant reduction, and $E_{tol}$ is the matrix energy threshold set up to construct the lower dimensional space; in our case, the energy tolerance is set up so that 4 LDA dimensions are utilized. Due to the curse of dimensionality, that is, the probability of sampling in the interior of a *n*-sphere that is inscribed in a *n*-dimensional hyper-prism approaches zero for n>10 [54,55].

This result also suggests that the correct reduced basis should not have more than 10 dimensions in an isotropic search space; therefore, the classification is limited to up to 10 classes. Nevertheless, the uncertainty space in linear inverse problems has an anisotropic character due to the ill-conditioning of the corresponding linear system. Therefore, the effective number of dimensions to be sampled is even lower.

Finally, the LDA reduced basis set is completed by adding a high frequency (HF) term, which is the model with the lowest energy, and projecting it into the LDA basis set as follows:

(8) $$v_{d+1}=m_{BEST}-\left(\;\mu +V_{d}a_{k}\right)$$

Including the high frequency term is crucial for a successful protein model reconstruction in Cartesian coordinates after the regularized LDA sampling. The combination of this high frequency term and the forward model calculations makes possible optimal protein reconstruction in the reduced basis. The HF term serves to span high frequency details of the reconstruction, helping to decrease the energy of the template.

### 2.2. Protein Modelling

The protein tertiary structure problem is performed here with the aid of the Bioshell package [56,57,58,59]. In essence, the problem concerns the optimization of the protein energy function, given the atom coordinates provided by the aligned templates as variables. Generally speaking, the number of input variables exceeds by far the number of protein templates utilized to model the protein 3D structure; therefore, the problem is deemed ill-posed. The modelling, as discussed in the Introduction, is not very different to classical and global optimization approaches, machine learning, and deep sampling. Normally, optimizations methods try to find a global energy optimum in a high dimensional space.

As mentioned in the previous subsection, a dDFIRE energy function and a GB/SA solvation model were utilized. The protein energy is determined by the contribution of those interactions. Predicting the protein tertiary structure consists of finding a protein model $m_{p}$ that minimizes the value of energy [60]. Mathematically,

(9) $$E\left(m\right):{\mathbb{R}}^{n}\to \mathbb{R}:m_{p}=\underset{m\in M}{\min}E\left(m\right)$$

where $m_{p}$ is the matrix containing the atom coordinates that minimizes the protein energy. Since it is a highly dimensional function, the energy landscape is intricate and complex. Mathematically, the native backbone structure satisfies the condition $\nabla E\left(m_{p}\right)=0$. As a consequence, it is possible to find a set of protein templates that are below a certain tolerance, are within the neighborhood of $m_{p}$, and that can be approximated by a hyper-quadric as follows:

(10) $$\frac{1}{2}{\left(m-m_{p}\right)}^{T}HE\left(m_{p}\right)\left(m-m_{p}\right)\leq E_{tol}-E\left(m_{p}\right)$$

where $HE\left(m_{p}\right)$ is the Hessian matrix evaluated at $m_{p}$. Due to the complexity of the energy function, high explorative global optimization methods are required in order to avoid getting trapped in flat curvilinear–elongated valleys [61,62]. In this paper, we utilize a particle swarm optimizer, a family member known as RR-PSO to sample the energy function in the reduced space [63].

### 2.3. Optimization of the Protein Energy Function

The particle swarm algorithm defines a prismatic space of admissible protein models, that is,

(11) $$l_{j\;}\leq a_{j\;}\leq u_{j\;\;}\;\;j=1,\;n_{size\;}$$

where $l_{j},u_{j}$ are the lower and upper limits for the j-th coordinate for each model, respectively, and $n_{size}$ is the size of the swarm. In this case, the order relation ≤ has to be interpreted component-wise.

In our case, the algorithm samples over the reduced base spanned by the regularized LDA reduced basis set. In the algorithm, each particle (model) has its own position in the search space while the velocity of the particle corresponds to the perturbations of atomic coordinates performed to explore the search space in the reduced basis. PSO has been confirmed as a good candidate to sample the alternate states by Fernández-Martínez et al. [64,65,66]. As an evolutionary sampling algorithm, it performs a deep sampling in order to find a protein model that satisfies the condition $E\left({\hat{m}}_{k}\right)\leq E_{tol}$. The sampled model must be reconstructed again in the original atom space in order to evaluate the atom coordinates, energy and forces.

### 2.4. Protein Refinement via Singular Value Decomposition

Once the PSO sampling is performed, there is still room for further improvement of the protein structure. We utilize a simple and fast refinement algorithm employing singular value decomposition, proposed by Alvarez-Machancoses et al. [67]. Building up a reduced search space via SVD aids in regularizing the inverse problem and finds the atom coordinates that minimize protein free-energy. The refinement is also carried with PSO over a reduced search space, provided by the obtained eigenvalues from the SVD according to Equation (7), where μ is the mean (it could be null) and $V_{d}$ is provided by the SVD.

The idea is similar to the regularized LDA model reduction; it consists of formatting the protein in a matrix format, ${\hat{m}}_{k}\in M\left(3,n_{\mathrm{atoms}}\right)$, where each column corresponds to the [x, y, z] coordinates of each atom. Then, the SVD factorization yields

(12) $${\hat{m}}_{k}\;=\;U\Sigma V^{T}=\sum_{k=1}^{3}\alpha_{k}u_{k}v_{k}^{T}$$

where U, V are orthogonal matrices whose column vectors are, respectively, $u_{k}\;\mathrm{and}\;v_{k}^{T},$ and Σ is the SVD of ${\hat{m}}_{k}$, containing 3 non-null singular values $\left(\alpha_{1},\alpha_{2},\alpha_{3}\right)$. The refinement is performed over the reduced basis $u_{k}v_{k}^{T}$, which contains only three components; therefore, in this reduced basis set, the protein ${\hat{m}}_{k}$ has only these three coordinates. Once the reduced basis set is defined, any other protein model will be spanned as a unique linear combination as ${\hat{m}}_{new}=\overset{3}{\underset{k=1}{\sum }}\beta_{k}u_{k}v_{k}^{T}$, and the reduced coordinates $\left(\beta_{1},\beta_{2},\beta_{3}\right)$ are obtained via PSO refinement.

## 3. Results

### 3.1. Overview of Computational Experiments

**Figure 2:**
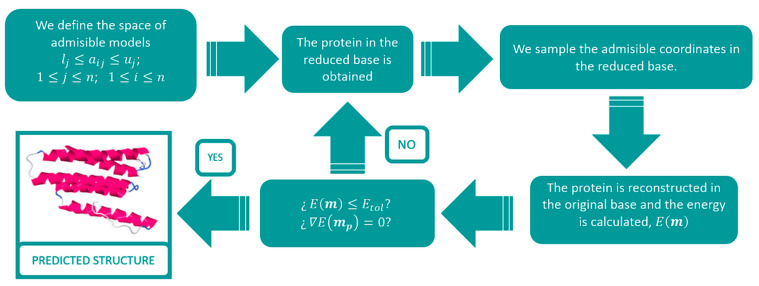
Overview of the protein modelling via regressive–regressive particle swarm optimization (RR-PSO).

The selection of protein samples was performed randomly, and the preselection of the templates for each protein was carried out according to energetic considerations. The idea was to consider all decoys that could yield to a plausible native structure model while being capable of sampling different backbone conformations (equivalent models). To accomplish this, each template was evaluated according to the energy. Each protein benchmark contained high- and low-quality templates. Consequently, in order to consider the best possible templates, while expanding all the possible protein conformations within the neighborhood of the native structure, we selected a cut-off corresponding to the 30th percentile. This number allowed us to expand/sample all the possible conformational differences while obtaining a good prediction of the native structure. In this sense, further restricting the cut-off will yield to a small prediction improvement, but fewer equivalent protein models will be sampled (see Figure 2 for a general flowchart of the Protein Tertiary Structure Prediction algorithm with model reduction techniques). After selecting the templates, a regularized LDA was utilized to divide the selected decoys in four classes, and, later on, we superimposed the templates to the best decoy. Consequently, the sampling of the energy function was carried out over five dimensions. The RR-PSO algorithm was utilized with a swarm of 40 particles and 50 iterations. Generally speaking, the RR-PSO algorithm works efficiently with a swarm size of 30–40, that is, a high explorative character without compromising the optimization. Once the optimum structure was found, the protein coordinates were reduced in three dimensions corresponding to the three eigenvalues, followed by an additional RR-PSO, which converged to the final predicted structure.

### 3.2. Template Selection and Protein Model Reduction

In this section we show the application of the LDA/SVD–PSO algorithm to the prediction of a set of proteins that were used as targets in past CASP experiments. The native structures of these proteins are known, are deposited in the PDB, and were solved through experimental methods such as NMR or X-ray crystallography. Therefore, all the detailed information about the protein’s structure, dynamics, and binding of nucleotides or other molecules is completely known.

The proteins that were modeled are summarized in Table 1. As mentioned, native structures were obtained via the Protein Data Bank and the templates were extracted from www.predictioncenter.org.

**Figure 3:**
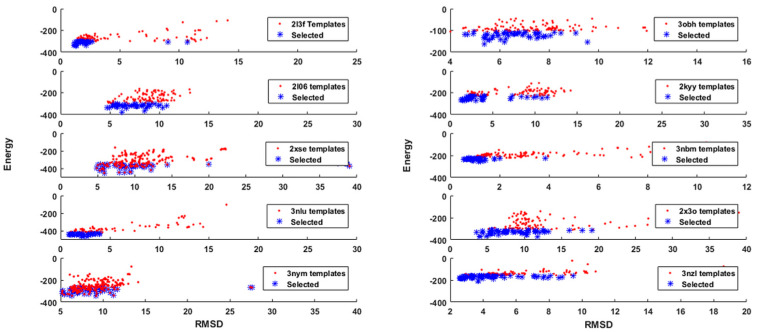
Template energy and energy selection.

Figure 3 shows the evaluated template energy with respect to the root mean squared distance to the native structure for the first 10 proteins (the rest can be found in the Supplementary Materials). Within the total amount of templates, it could be observed that both high-quality and low-quality templates were included. The idea was to consider the best protein templates by selecting them with an energy cut-off. In this sense, we considered all those proteins templates whose energy was within the 30th percentile (templates represented with the blue marker). The calculation of this percentile does not preclude the knowledge of the energy of the native structure. By applying this criterion, it is possible to consider all those decoys that fall within the neighborhood of the protein native structure according to its energy, while conserving a high variability of RMSD, which helps us evaluate a wider range of protein structural conformations.

**Figure 4:**
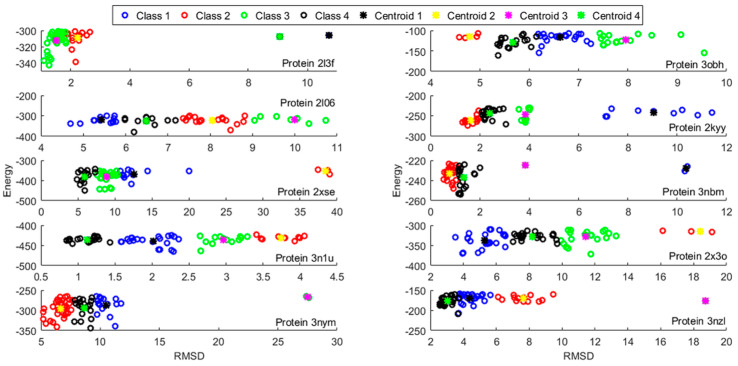
Template classification based on energy and structural considerations.

By zooming in on each subplot and focusing on the templates within the 30th percentile, we performed the classification and observed that four classes was a fair number, which served to span almost the entire variability for the proteins selected. Figure 4 shows the templates separated by classes for the first 10 proteins (the rest can be found in the Supplementary Materials), where the centroid of each class is also superimposed in this figure.

**Figure 5:**
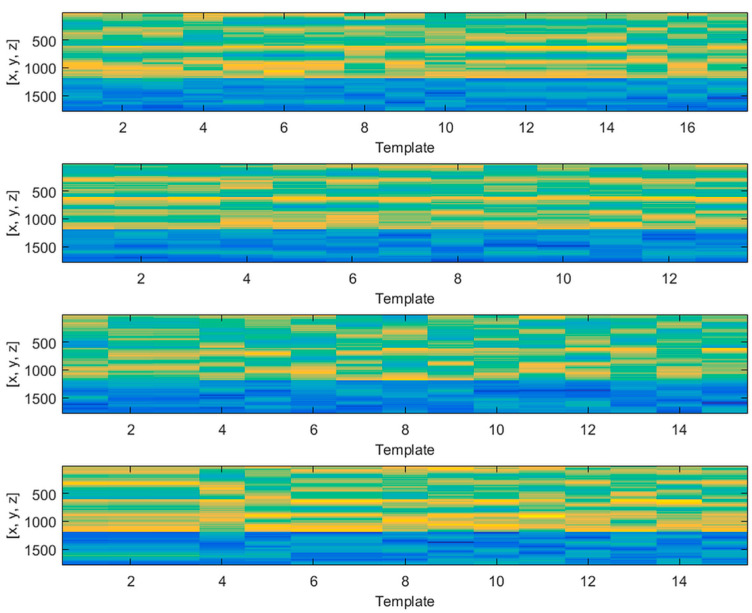
Example of protein classification. Protein 3obh class division and intraclass structural similarity.

In addition, we show the protein 2l3f class separation in Figure 5, where the *y* axis represents the coordinate value of each atom and the *x* axis corresponds to each decoy (basis set component). In other words, Figure 4 shows the unit basis vectors to construct the low dimensional subspace (4-dimensional) of the original backbone structure where the PSO optimization takes place. This supposes a drastic dimensionality reduction from $3n_{atoms}$ to 4.

**Figure 6:**
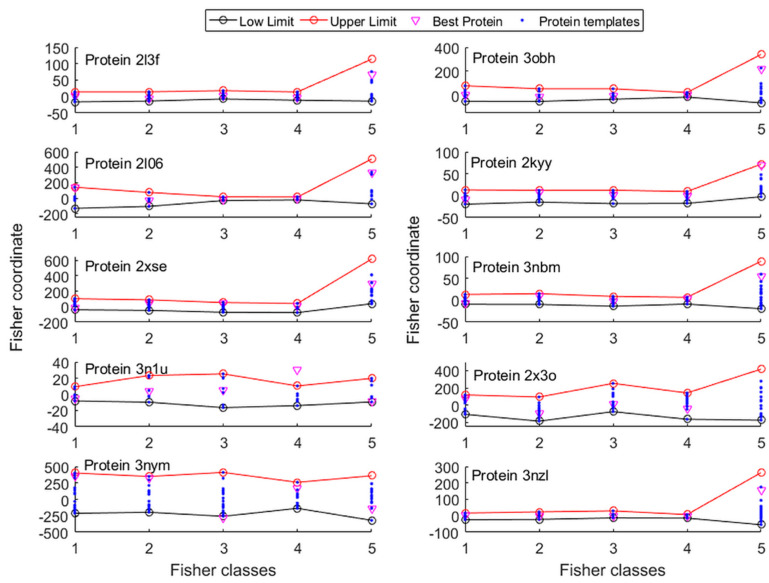
Protein search space constructed via L2-regularized LDA.

Figure 6 represents the search space for the first 10 proteins utilized to carry out the PSO sampling (further information about the search space utilized in the rest of the experiments can be found in the Supplementary Materials). The search space was defined by projecting the proteins within each class over each class vector and finding the minimum and maximum coordinates. This search space is indicative and could be further expanded if needed.

### 3.3. Protein Model Optimization and Refinement

Over the defined search spaces, a PSO optimization was carried out. For each protein case, PSO sampling was performed with a swarm composed of 40 particles and 50 iterations. To perform this task, the family member, RR-PSO was selected, whose exploration capabilities were monitored in order to ensure that a proper exploration of the reduced LDA basis was performed. Monitoring of the PSO sampling was carried out by defining the median dispersion of each swarm particle with respect to the center of gravity. The distance was normalized in such a way that the first iteration corresponded to a 100% dispersion. When the median dispersion fell below 3%, it was considered that the PSO algorithm had collapsed towards a global optimum. When this collapse happens, all the particles of the same iteration are considered as a unique particle in the posterior sampling; that way, these models are not overrepresented due to this numerical artefact.

Table 2 shows the details of the computations performed with LDA–SVD and RR-PSO. With only 50 iterations and a swarm of 40 particles it was sufficient to perform a deep sampling and achieve the global optimum over the defined search space. It is also worth mentioning that the sampling performance was strongly dependent on the protein energy function and the search space.

Once the algorithm provides particle dispersion below 3% and no further improvement in the energy is observed, it is possible to conclude that a global optimum is found. The predicted structures are summarized in Table 2. We present the quantitative assessment of the predicted structures via the RMSD, alongside the predictions carried by other two established methodologies, such as Zhang server and Rosetta server. As can be seen, the obtained results suggest that there is a statistically significant similarity between the predicted structures (Table 3).

Further nuance about the predicted protein structures is given by showing the native backbone structure and the predicted one superimposed, as shown in Supplementary Figures S1–S30.

## 4. Discussion

By merging energy-based modelling with sampling along regularized LDA coordinates, we are capable of overcoming the two main drawbacks of energy-based methods of comparative models, which are the very intricate energy landscape sampled and the inaccuracy of the force fields. In this sense, it is possible to utilize energy and force field models with lower resolution. The sampling is generally greatly improved because the LDA coordinates represent concerted movements of the chain and, in addition, represent different backbone conformations of a given protein, that is, different evolutionary directions. Since the model dimensionality is reduced drastically, problems associated with the energy function inaccuracy are also reduced and partially overcome, a result that is aligned with Quian et al. [67].

The model results indicate that the LDA/SVD–PSO is capable of converging to the optimum structure robustly, with low sensitivity to alignment errors. However, in those cases where the structure is very complex, a large ill-conditioned matrix of templates is obtained that yields to highly regularized LDA coordinates. In these cases, it is of utmost importance to constrain the number of templates to those with the lowest-energy. In this sense, as a future work it would be interesting to include iterative alignment and model evaluation methods alongside the model reduction with LDA in order to perform a higher resolution prediction.

The fact that this methodology classifies the templates based on “a priori” information, it would be interesting to expand it and generalize it to other fields within proteomics, such as in protein–protein docking and quaternary structure prediction, since plausible conformations could be represented by different reduced LDA coordinates.

## 5. Conclusions

In this research paper, an algorithm that corresponds to the category of template-based modeling is presented. In general, the algorithm uses LDA in combination with SVD as mathematical techniques to perform model reduction in a template-based modelling general methodology. The main idea is to obtain a different perspective with respect to other similar methods such as Alvarez-Machancoses et al. [43], which uses PCA in combination with PSO, or Baker et al., which uses PCA and a simplex and Powell method optimization [68].

As outlined, the algorithm is intended to create a low-dimensional space in order to apply an energy optimization procedure via particle swarm optimization. The low-dimensional space is constructed with a regularized linear discriminant analysis in order to make the algorithm robust enough and overcome possible singularity problems when dealing with high-dimensional data. The optimization over the reduced space is carried out with the RR-PSO algorithm, which combines strong optimization and exploration capabilities. The predicted optimal structure corresponds to the nonlinear equivalent region lower than a certain energy threshold. Since this predicted structure may not correspond exactly to the native backbone structure, further refinement utilizing a simple and fast SVD refinement algorithm is carried out. This last step involves optimization and uncertainty analysis via PSO in four dimensions and serves to improve the results provided by LDA–PSO. The present algorithm is capable of alleviating the ill-posed character of this highly-dimensional optimization problem when a protein is projected over the reduced search space, and it is computationally very efficient.

The source code is available from us.

## Figures and Tables

**Table 1:** Summary of the protein selected and the number of templates available alongside the class division.

Protein (CASP Code)	Number of Residues	Number of Templates	Number of Classes
2l3f (T0545)	166	185	4
3obh (T0551)	82	199	4
2l06 (T0555)	155	182	4
2kyy (T0557)	153	183	4
2xse (T0561)	170	180	4
3nbm (T0580)	108	195	4
3n1u (T0635)	191	181	4
2x3o (T0637)	240	194	4
3nym (T0639)	128	206	4
3nzl (T0643)	82	178	4
4pqx (T0760)	217	94	4
4q69 (T0770)	462	100	4
4qdy (T0780)	227	103	4
4l4w (T0790)	295	107	4
4qrk (T0800)	220	277	4
Q6MI90_BDEBA (T0810)	383	164	4
VCID6010 (T0820)	140	333	4
5f15 (T0830)	575	225	4
4gt8 (T0840)	669	96	4
U1 Protein (T0850)	190	268	4
5d9g (T0864)	246	264	4
5j5v (T0870)	323	268	4
1ctf (T0880)	787	321	4
5t87 (T0885)	116	122	4
3k1e (T0890)	125	321	4
5aot (T0900)	106	255	4
6c0t (T0910)	347	105	4
5ere (T0920)	568	91	4
5sy1 (T0930)	149	187	4
1o6d (T0940)	163	259	4

**Table 2:** Details of the computational experiments performed with the methodology presented in this paper via LDA–SVD and PSO.

Protein (CASP Code)	Number of Residues	Number of Classes	Reduced Basis Terms	Percentile of Decoys	Number of Iterations	Swarm Size	Energy Obtained
2l3f (T0545)	166	4	5	30	50	40	−343.86
3obh (T0551)	82	4	5	30	50	40	−163.42
2l06 (T0555)	155	4	5	30	50	40	−381.96
2kyy (T0557)	153	4	5	30	50	40	−152.77
2xse (T0561)	170	4	5	30	50	40	−449.50
3nbm (T0580)	108	4	5	30	50	40	−255.42
3n1u (T0635)	191	4	5	30	50	40	−369.47
2x3o (T0637)	240	4	5	30	50	40	−372.10
3nym (T0639)	128	4	5	30	50	40	−343.22
3nzl (T0643)	82	4	5	30	50	40	−210.34
4pqx (T0760)	217	4	5	30	50	40	−496.11
4q69 (T0770)	462	4	5	30	50	40	−992.46
4qdy (T0780)	227	4	5	30	50	40	−425.77
4l4w (T0790)	295	4	5	30	50	40	−598.56
4qrk (T0800)	220	4	5	30	50	40	−502.35
Q6MI90_BDEBA (T0810)	383	4	5	30	50	40	−902.65
VCID6010 (T0820)	140	4	5	30	50	40	−356.56
5f15 (T0830)	575	4	5	30	50	40	−1214.65
4gt8 (T0840)	669	4	5	30	50	40	−1115.98
U1 Protein (T0850)	190	4	5	30	50	40	−448.13
5d9g (T0864)	246	4	5	30	50	40	−545.61
5j5v (T0870)	323	4	5	30	50	40	−408.32
1ctf (T0880)	787	4	5	30	50	40	−398.39
5t87 (T0885)	116	4	5	30	50	40	−298.43
3k1e (T0890)	125	4	5	30	50	40	−561.94
5aot (T0900)	106	4	5	30	50	40	−208.01
6c0t (T0910)	347	4	5	30	50	40	−838.89
5ere (T0920)	568	4	5	30	50	40	−1229.68
5sy1 (T0930)	149	4	5	30	50	40	−1812.17
1o6d (T0940)	163	4	5	30	50	40	−627.02

**Table 3:** RMSDs predicted structures via LDA–SVD and particle swarm optimization compared to Rosetta and Zhang servers.

Protein (CASP Code)	RMSD LDA–SVD	RMSD Zhang Server	RMSD Rosetta Server
2l3f (T0545)	1.27	2.17	2.38
3obh (T0551)	5.30	2.75	2.65
2l06 (T0555)	6.16	2.99	3.20
2kyy (T0557)	1.16	2.54	2.08
2xse (T0561)	5.88	3.01	3.09
3nbm (T0580)	1.16	1.80	1.37
3n1u (T0635)	1.31	0.74	1.08
2x3o (T0637)	5.18	2.44	2.61
3nym (T0639)	6.70	2.74	2.11
3nzl (T0643)	3.67	2.72	2.75
4pqx (T0760)	2.73	2.93	3.21
4q69 (T0770)	5.01	4.53	4.47
4qdy (T0780)	3.12	2.97	2.93
4l4w (T0790)	3.81	4.96	4.48
4qrk (T0800)	12.25	7.25	9.80
Q6MI90_BDEBA (T0810)	13.85	8.30	14.76
VCID6010 (T0820)	12.97	9.32	14.75
5f15 (T0830)	26.57	20.77	11.15
4gt8 (T0840)	3.03	2.71	4.99
U1 Protein (T0850)	3.65	3.48	4.03
5d9g (T0864)	2.81	2.58	2.10
5j5v (T0870)	17.12	12.67	11.84
1ctf (T0880)	6.66	8.69	7.59
5t87 (T0885)	2.87	3.92	2.93
3k1e (T0890)	8.93	3.99	8.02
5aot (T0900)	3.32	7.63	4.67
6c0t (T0910)	1.74	1.58	1.83
5ere (T0920)	2.38	2.40	2.32
5sy1 (T0930)	4.56	3.27	4.25
1o6d (T0940)	4.13	3.71	3.07

## Supplementary Materials

The following are available online. Supplementary Figures S1–S30: The set of 30 Figures S1–S10 showing the backbone overlap of predicted structure and the native structure for 2kyy (Figure S1), 2l3f (Figure S2), 2l06 (Figure S3), 2x3o (Figure S4), 2xse (Figure S5), 3n1u (Figure S6), 3nbm (Figure S7), 3nym (Figure S8), 3nzl (Figure S9), 3obh (Figure S10), 4pqx (Figure S11), 4q69 (Figure S12), 4qdy (Figure S13), 4l4w (Figure S14), 4qrk (Figure S15), Q6MI90_BDEBA (Figure S16), VCID6010 (Figure S17), 5f15 (Figure S18), 4gt8 (Figure S19), U1 Protein (Figure S20), 5d9g (Figure S21), 5j5v (Figure S22), 1ctf (Figure S23), 5t87 (Figure S24), 3k1e (Figure S25), 5aot (Figure S26), 6c0t (Figure S27), 5ere (Figure S28), 5sy1 (Figure S29) and 1o6d (Figure S30). Supplementary Material to Figure 3 S1–S20: 4pqx (Figure 3—S1), 4q69 (Figure 3—S2), 4qdy (Figure 3—S3), 4l4w (Figure 3—S4), 4qrk (Figure 3—S5), Q6MI90_BDEBA (Figure 3—S6), VCID6010 (Figure 3—S7), 5f15 (Figure 3—S8), 4gt8 (Figure 3—S9), U1 Protein (Figure 3—S10), 5d9g (Figure 3—S11), 5j5v (Figure 3—S12), 1ctf (Figure 3—S13), 5t87 (Figure 3—S14), 3k1e (Figure 3—S15), 5aot (Figure 3—S16), 6c0t (Figure 3—S17), 5ere (Figure 3—S18), 5sy1 (Figure 3—S19) and 1o6d (Figure 3—S20). Supplementary Material to Figure 4 S1–S20: 4pqx (Figure 4—S1), 4q69 (Figure 4—S2), 4qdy (Figure 4—S3), 4l4w (Figure 4—S4), 4qrk (Figure 4—S5), Q6MI90_BDEBA (Figure 4—S6), VCID6010 (Figure 4—S7), 5f15 (Figure 4—S8), 4gt8 (Figure 4—S9), U1 Protein (Figure 4—S10), 5d9g (Figure 4—S11), 5j5v (Figure 4—S12), 1ctf (Figure 4—S13), 5t87 (Figure 4—S14), 3k1e (Figure 4—S15), 5aot (Figure 4—S16), 6c0t (Figure 4—S17), 5ere (>Figure 4—S18), 5sy1 (Figure 4—S19) and 1o6d (Figure 4—S20). Supplementary Material to Figure 6 S1–S20: 4pqx (>Figure 6—S1), 4q69 (Figure 6—S2), 4qdy (Figure 6—S3), 4l4w (Figure 6—S4), 4qrk (Figure 6—S5), Q6MI90_BDEBA (>Figure 6—S6), VCID6010 (Figure 6—S7), 5f15 (Figure 6—S8), 4gt8 (Figure 6—S9), U1 Protein (Figure 6—S10), 5d9g (Figure 6—S11), 5j5v (Figure 6—S12), 1ctf (Figure 6—S13), 5t87 (Figure 6—S14), 3k1e (igure 6—S15), 5aot (Figure 6—S16), 6c0t (Figure 6—S17), 5ere (Figure 6—S18), 5sy1 (Figure 6—S19) and 1o6d (Figure 6—S20).
