# Supplementary figures and images for: Prediction of Protein Tertiary Structure via Regularized Template Classification Techniques
Source: Molecules. 2020 May 26;25(11):2467. doi: 10.3390/molecules25112467 (PMC7321371; doi:10.3390/molecules25112467)

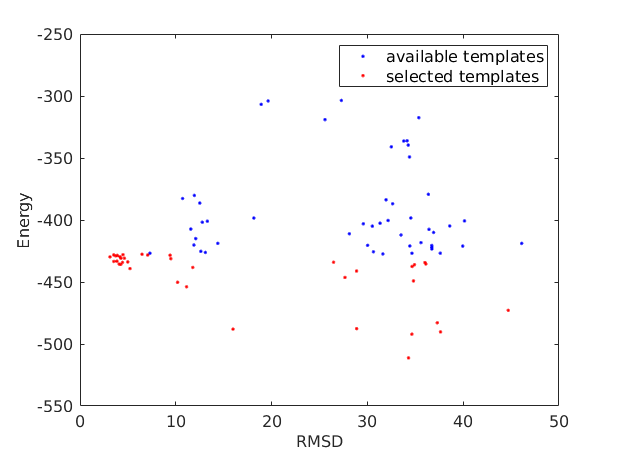

Supplement: Supplementary file 1 [file molecules-25-02467-s001.zip › Figure 3 - Supplementary Material/Figure 3 - S1 - 4pqx .tif]

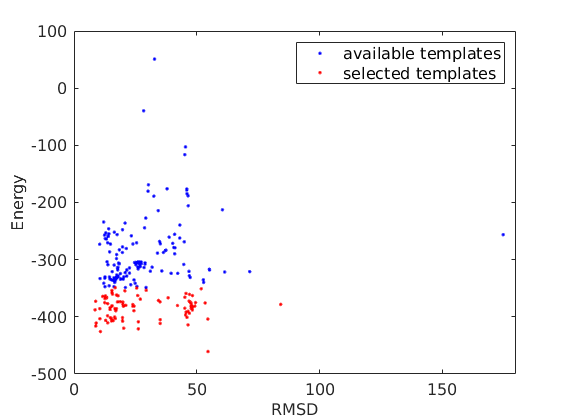

Supplement: Supplementary file 1 [file molecules-25-02467-s001.zip › Figure 3 - Supplementary Material/Figure 3 - S10 - U1.tif]

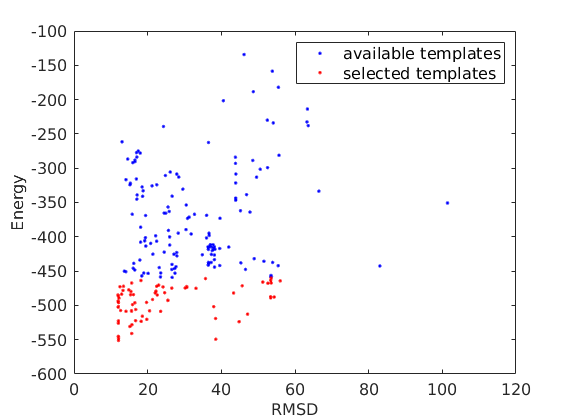

Supplement: Supplementary file 1 [file molecules-25-02467-s001.zip › Figure 3 - Supplementary Material/Figure 3 - S11 - 5d9g.tif]

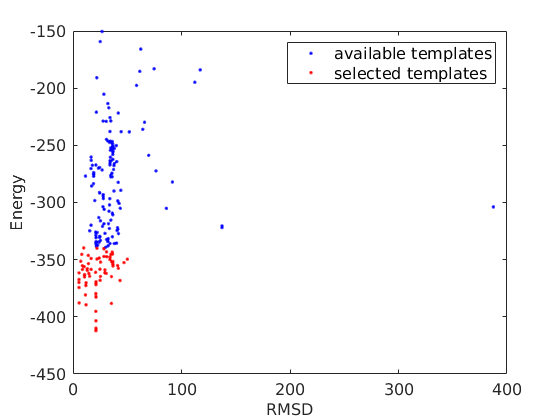

Supplement: Supplementary file 1 [file molecules-25-02467-s001.zip › Figure 3 - Supplementary Material/Figure 3 - S12 - 5j5v.tif]

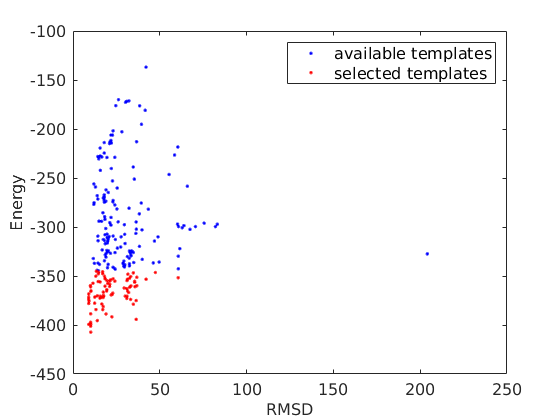

Supplement: Supplementary file 1 [file molecules-25-02467-s001.zip › Figure 3 - Supplementary Material/Figure 3 - S13 - 1ctf.tif]

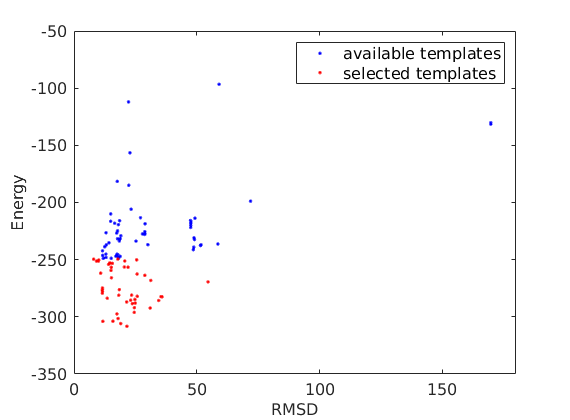

Supplement: Supplementary file 1 [file molecules-25-02467-s001.zip › Figure 3 - Supplementary Material/Figure 3 - S14 - 5t87.tif]

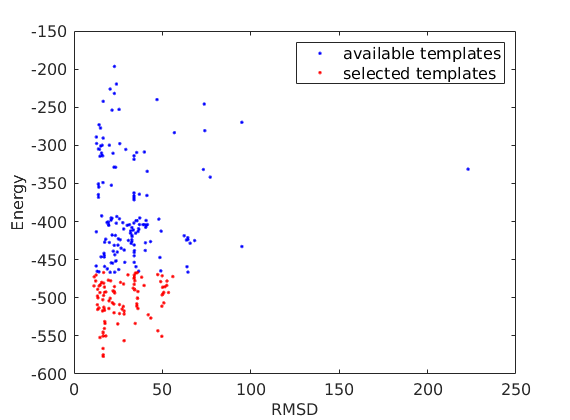

Supplement: Supplementary file 1 [file molecules-25-02467-s001.zip › Figure 3 - Supplementary Material/Figure 3 - S15 - 3k1e.tif]

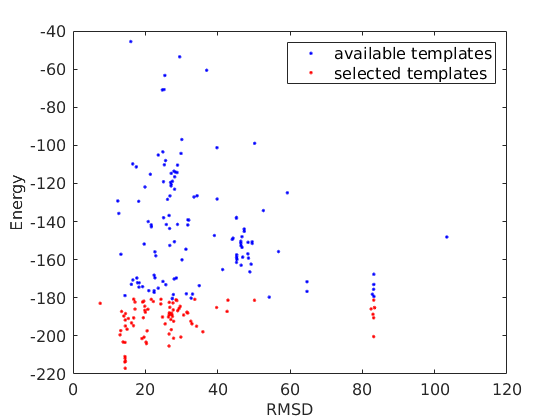

Supplement: Supplementary file 1 [file molecules-25-02467-s001.zip › Figure 3 - Supplementary Material/Figure 3 - S16 - 5aot.tif]

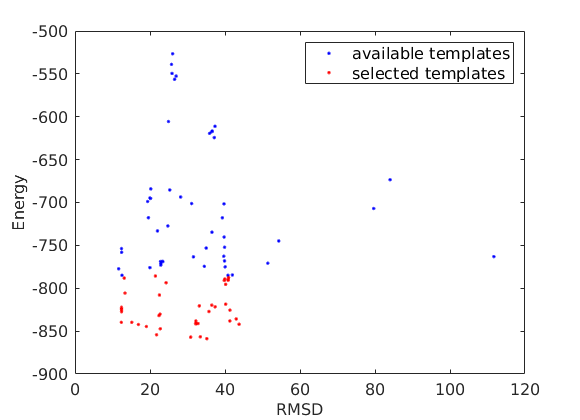

Supplement: Supplementary file 1 [file molecules-25-02467-s001.zip › Figure 3 - Supplementary Material/Figure 3 - S17 - 6c0t.tif]

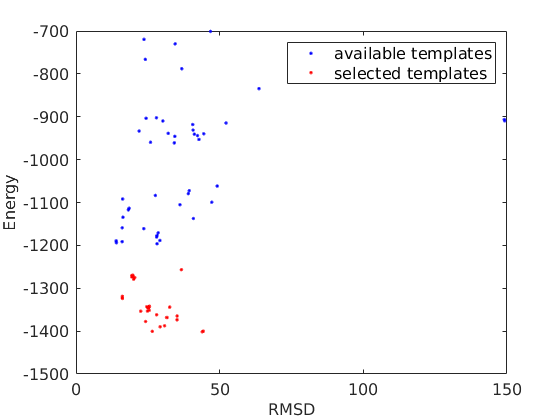

Supplement: Supplementary file 1 [file molecules-25-02467-s001.zip › Figure 3 - Supplementary Material/Figure 3 - S18 - 5ere.tif]

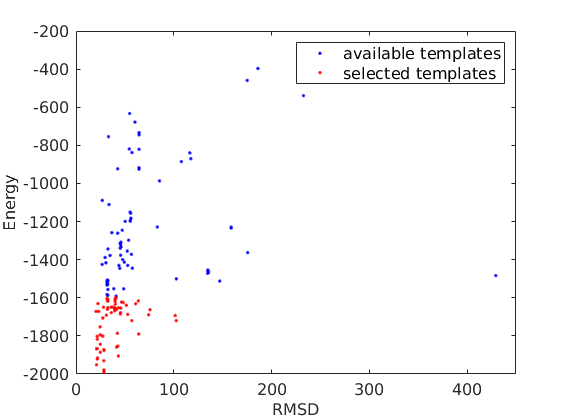

Supplement: Supplementary file 1 [file molecules-25-02467-s001.zip › Figure 3 - Supplementary Material/Figure 3 - S19 - 5sy1.tif]

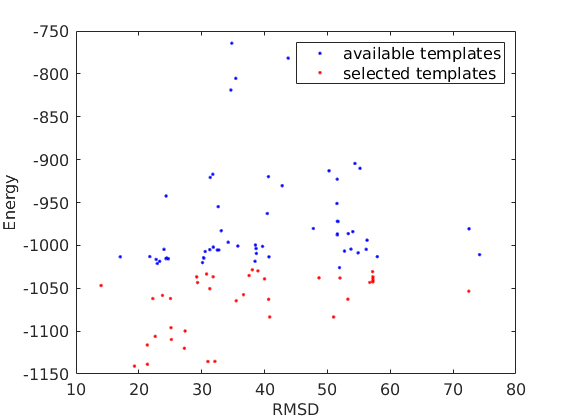

Supplement: Supplementary file 1 [file molecules-25-02467-s001.zip › Figure 3 - Supplementary Material/Figure 3 - S2 - 4q69.tif]

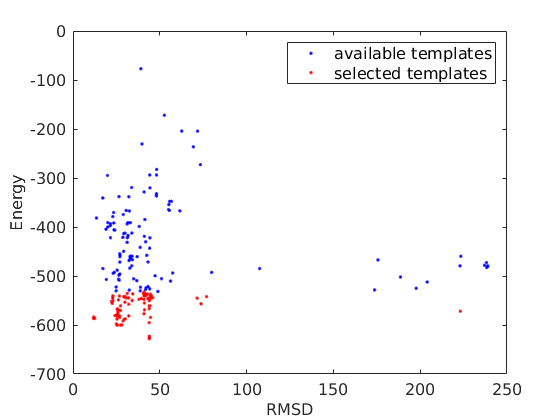

Supplement: Supplementary file 1 [file molecules-25-02467-s001.zip › Figure 3 - Supplementary Material/Figure 3 - S20 - 1o6d.tif]

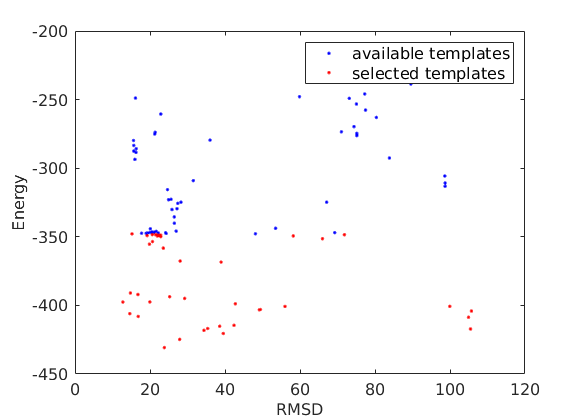

Supplement: Supplementary file 1 [file molecules-25-02467-s001.zip › Figure 3 - Supplementary Material/Figure 3 - S3 - 4qdy.tif]

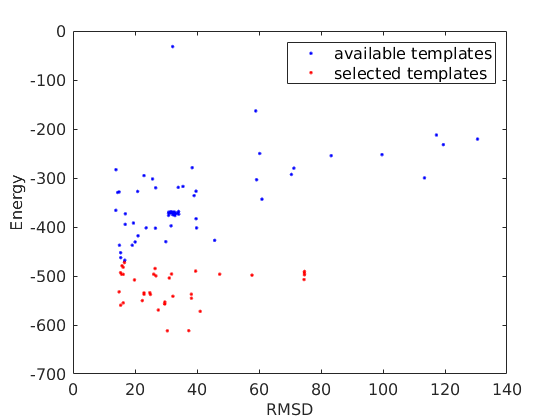

Supplement: Supplementary file 1 [file molecules-25-02467-s001.zip › Figure 3 - Supplementary Material/Figure 3 - S4 - 4l4w.tif]

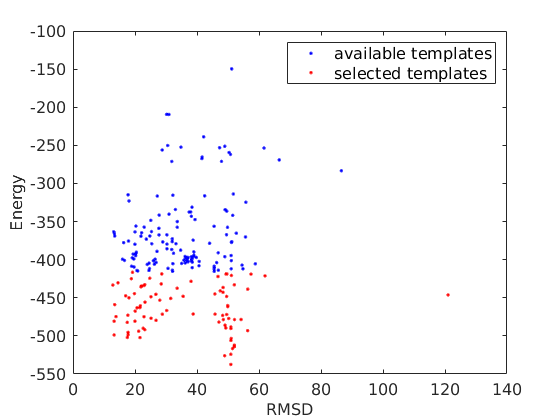

Supplement: Supplementary file 1 [file molecules-25-02467-s001.zip › Figure 3 - Supplementary Material/Figure 3 - S5 - 4qrk.tif]

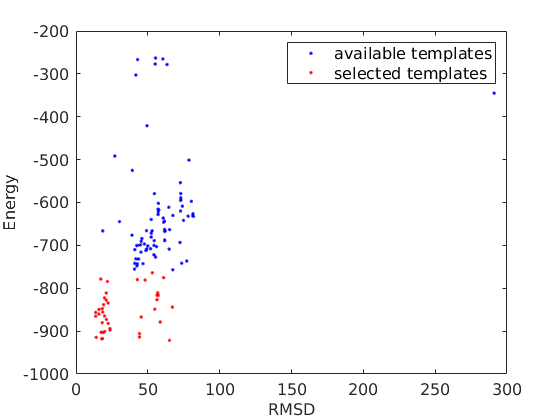

Supplement: Supplementary file 1 [file molecules-25-02467-s001.zip › Figure 3 - Supplementary Material/Figure 3 - S6 - Q6MI90_BDEBA.tif]

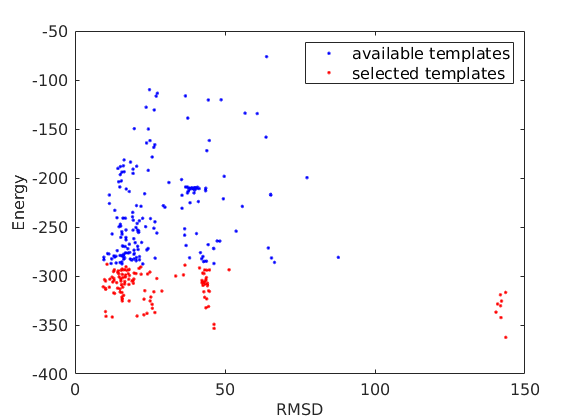

Supplement: Supplementary file 1 [file molecules-25-02467-s001.zip › Figure 3 - Supplementary Material/Figure 3 - S7 - VCID6010.tif]

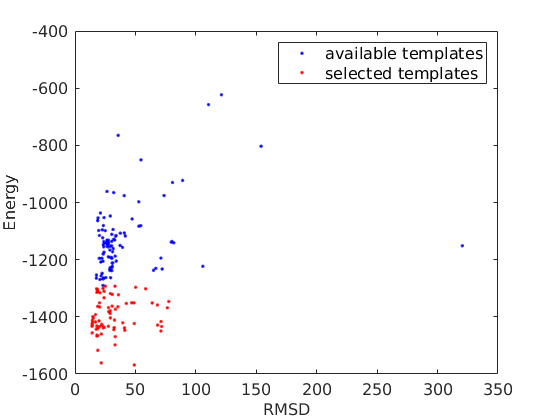

Supplement: Supplementary file 1 [file molecules-25-02467-s001.zip › Figure 3 - Supplementary Material/Figure 3 - S8 - 5f15.tif]

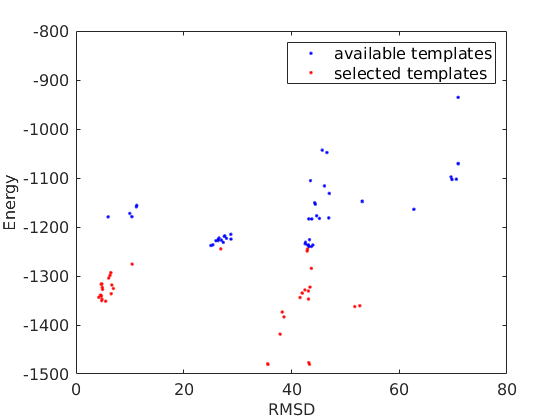

Supplement: Supplementary file 1 [file molecules-25-02467-s001.zip › Figure 3 - Supplementary Material/Figure 3 - S9 - 4gt8.tif]

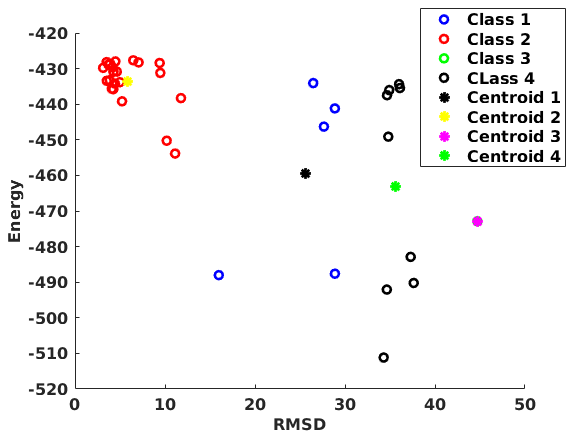

Supplement: Supplementary file 1 [file molecules-25-02467-s001.zip › Figure 4 - Supplementary Material/Figure 4 - S1 - 4pqx.tif]

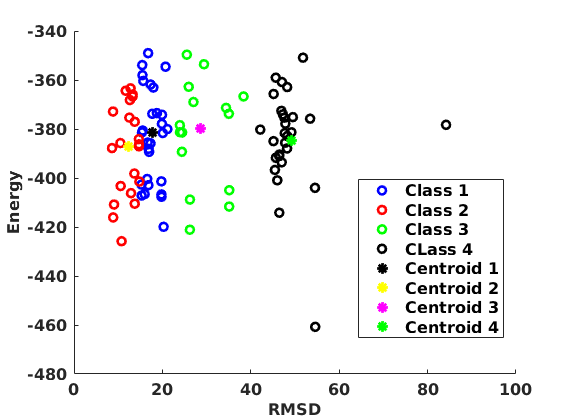

Supplement: Supplementary file 1 [file molecules-25-02467-s001.zip › Figure 4 - Supplementary Material/Figure 4 - S10 - U1.tif]

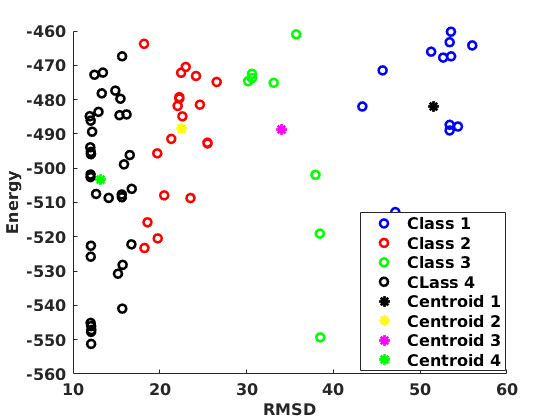

Supplement: Supplementary file 1 [file molecules-25-02467-s001.zip › Figure 4 - Supplementary Material/Figure 4 - S11 - 5d9g.tif]

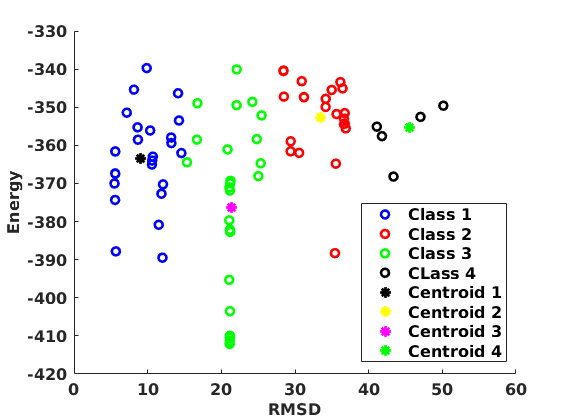

Supplement: Supplementary file 1 [file molecules-25-02467-s001.zip › Figure 4 - Supplementary Material/Figure 4 - S12 - 5j5v.tif]

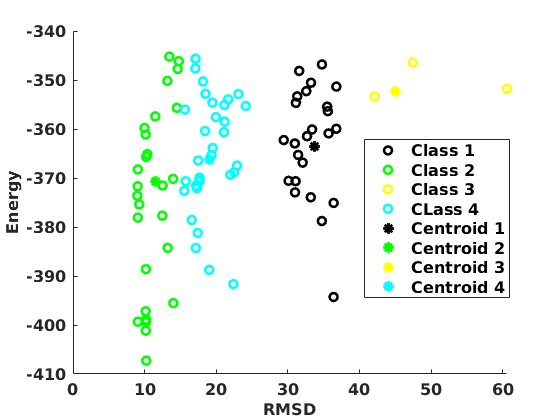

Supplement: Supplementary file 1 [file molecules-25-02467-s001.zip › Figure 4 - Supplementary Material/Figure 4 - S13 - 1ctf.tif]

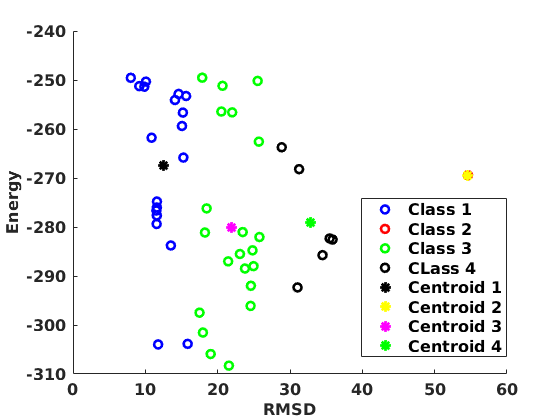

Supplement: Supplementary file 1 [file molecules-25-02467-s001.zip › Figure 4 - Supplementary Material/Figure 4 - S14 - 5t87.tif]

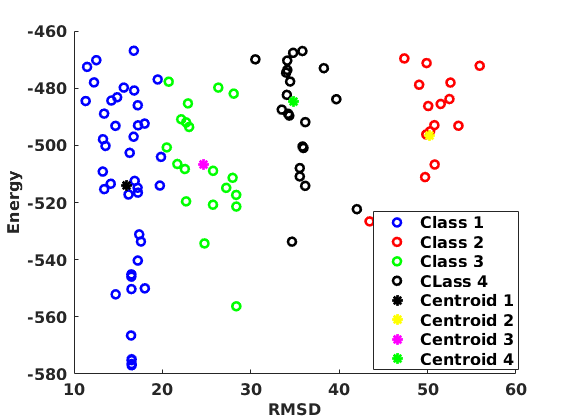

Supplement: Supplementary file 1 [file molecules-25-02467-s001.zip › Figure 4 - Supplementary Material/Figure 4 - S15 - 3k1e.tif]

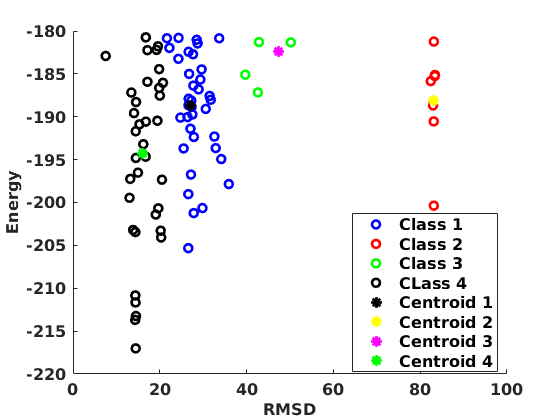

Supplement: Supplementary file 1 [file molecules-25-02467-s001.zip › Figure 4 - Supplementary Material/Figure 4 - S16 - 5aot.tif]

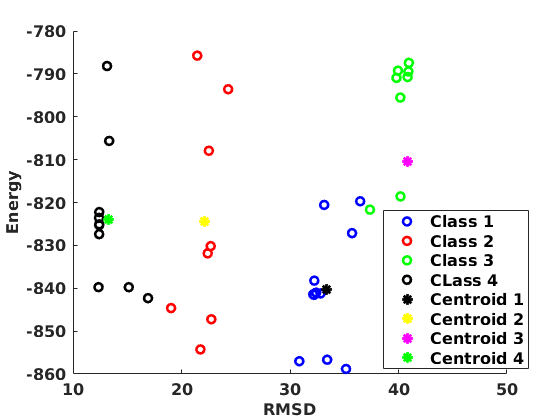

Supplement: Supplementary file 1 [file molecules-25-02467-s001.zip › Figure 4 - Supplementary Material/Figure 4 - S17 - 6c0t.tif]

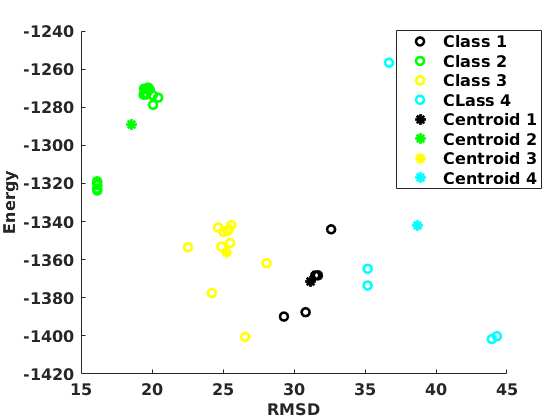

Supplement: Supplementary file 1 [file molecules-25-02467-s001.zip › Figure 4 - Supplementary Material/Figure 4 - S18 - 5ere.tif]

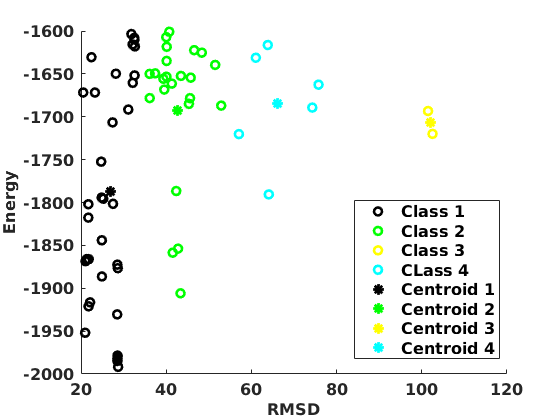

Supplement: Supplementary file 1 [file molecules-25-02467-s001.zip › Figure 4 - Supplementary Material/Figure 4 - S19 - 5sy1.tif]

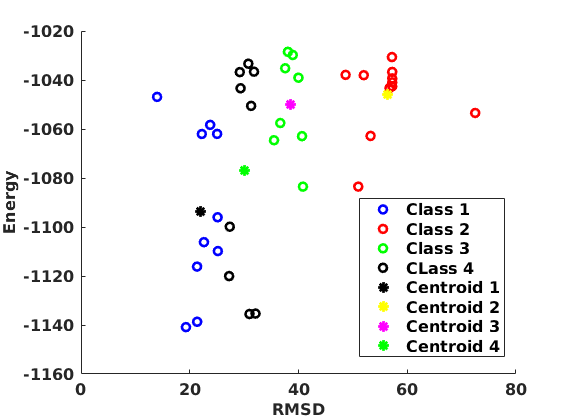

Supplement: Supplementary file 1 [file molecules-25-02467-s001.zip › Figure 4 - Supplementary Material/Figure 4 - S2 - 4q69.tif]

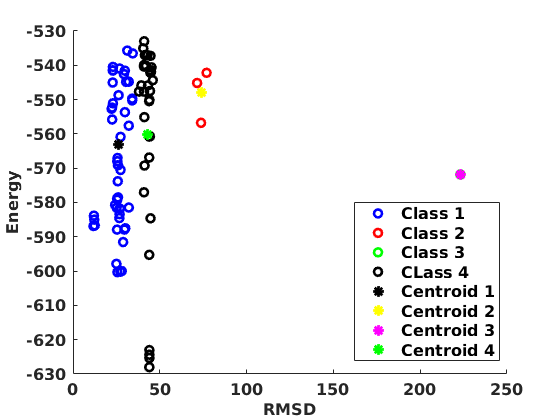

Supplement: Supplementary file 1 [file molecules-25-02467-s001.zip › Figure 4 - Supplementary Material/Figure 4 - S20 - 1o6d.tif]

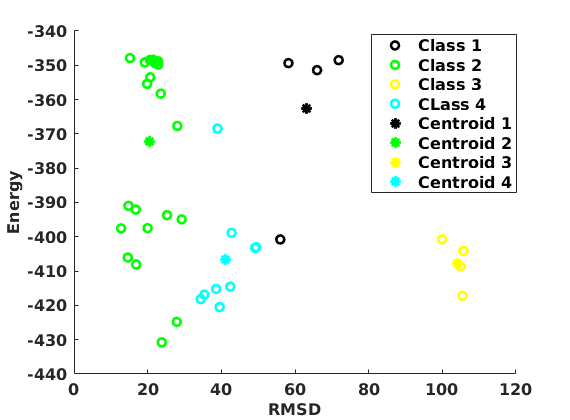

Supplement: Supplementary file 1 [file molecules-25-02467-s001.zip › Figure 4 - Supplementary Material/Figure 4 - S3 - 4qdy.tif]

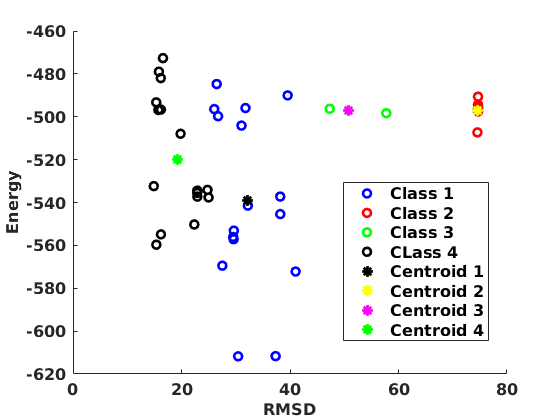

Supplement: Supplementary file 1 [file molecules-25-02467-s001.zip › Figure 4 - Supplementary Material/Figure 4 - S4 - 4l4w.tif]

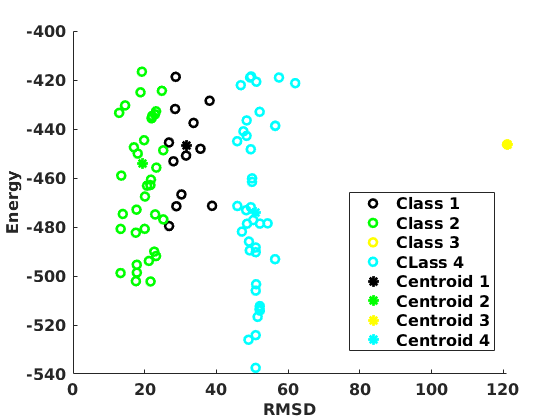

Supplement: Supplementary file 1 [file molecules-25-02467-s001.zip › Figure 4 - Supplementary Material/Figure 4 - S5 - 4qrk.tif]

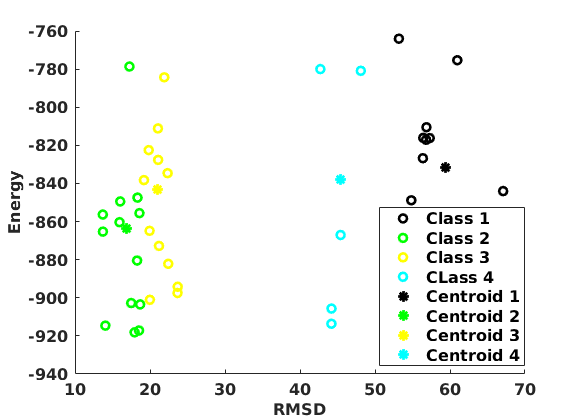

Supplement: Supplementary file 1 [file molecules-25-02467-s001.zip › Figure 4 - Supplementary Material/Figure 4 - S6 - Q6MI90_BDEBA.tif]

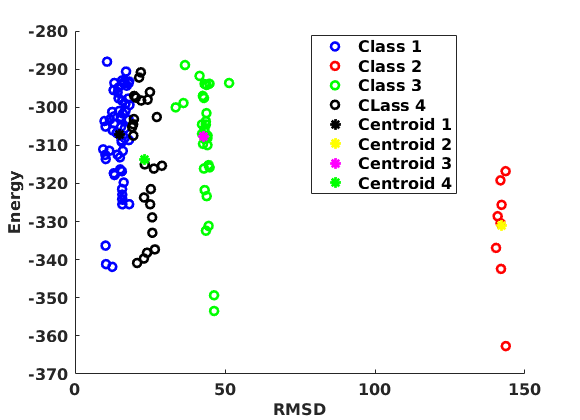

Supplement: Supplementary file 1 [file molecules-25-02467-s001.zip › Figure 4 - Supplementary Material/Figure 4 - S7 - VCID6010.tif]

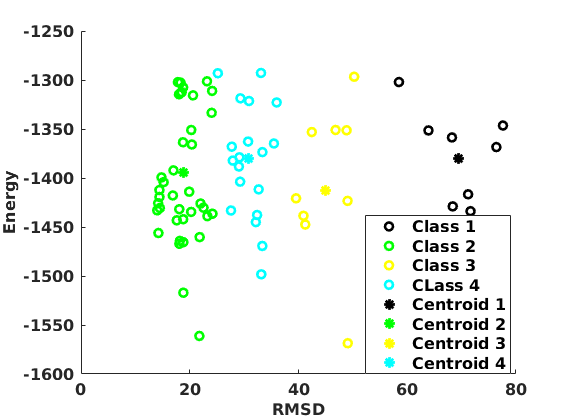

Supplement: Supplementary file 1 [file molecules-25-02467-s001.zip › Figure 4 - Supplementary Material/Figure 4 - S8 - 5f15.tif]

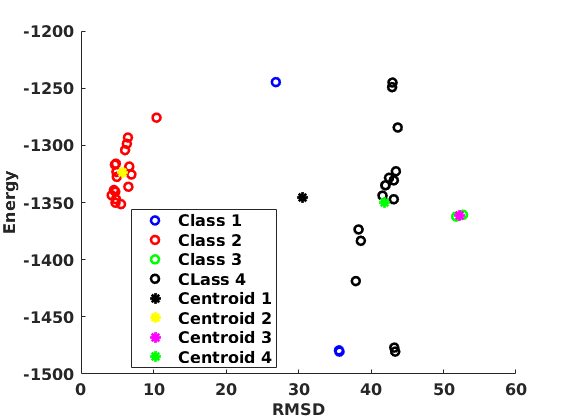

Supplement: Supplementary file 1 [file molecules-25-02467-s001.zip › Figure 4 - Supplementary Material/Figure 4 - S9 - 4gt8.tif]

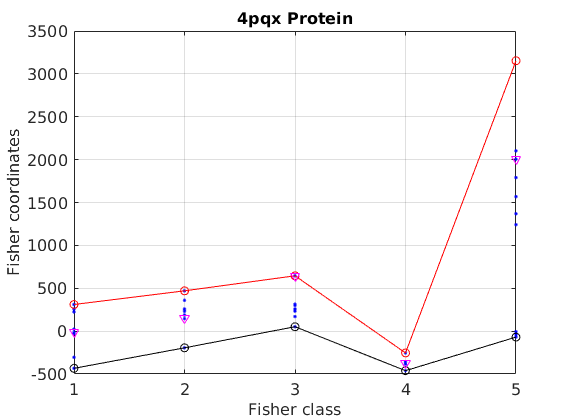

Supplement: Supplementary file 1 [file molecules-25-02467-s001.zip › Figure 6 - Supplementary Material/Figure 6 - S1 - 4pqx.tif]

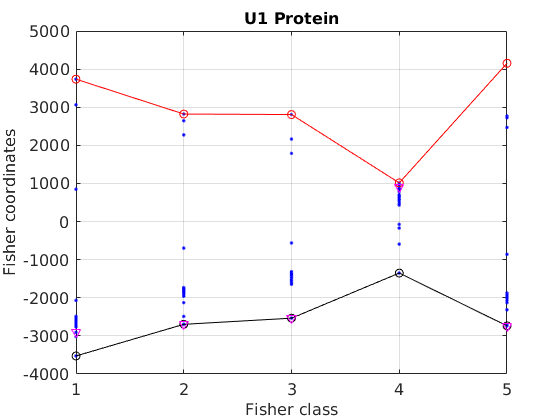

Supplement: Supplementary file 1 [file molecules-25-02467-s001.zip › Figure 6 - Supplementary Material/Figure 6 - S10 - U1.tif]

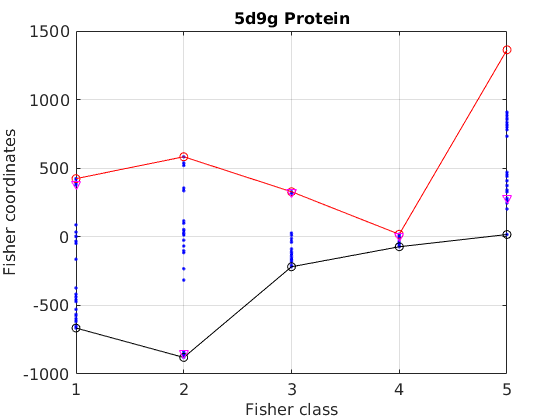

Supplement: Supplementary file 1 [file molecules-25-02467-s001.zip › Figure 6 - Supplementary Material/Figure 6 - S11 - 5d9g.tif]

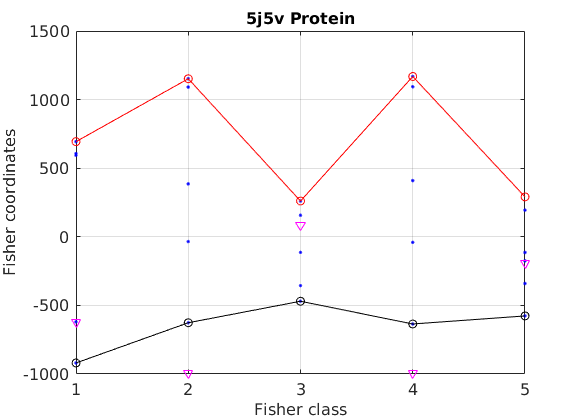

Supplement: Supplementary file 1 [file molecules-25-02467-s001.zip › Figure 6 - Supplementary Material/Figure 6 - S12 - 5j5v.tif]

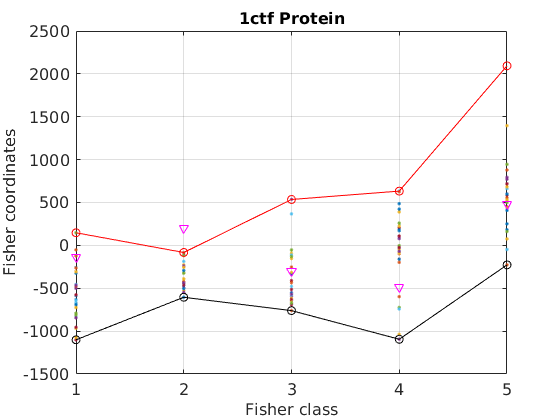

Supplement: Supplementary file 1 [file molecules-25-02467-s001.zip › Figure 6 - Supplementary Material/Figure 6 - S13 - 1ctf.tif]

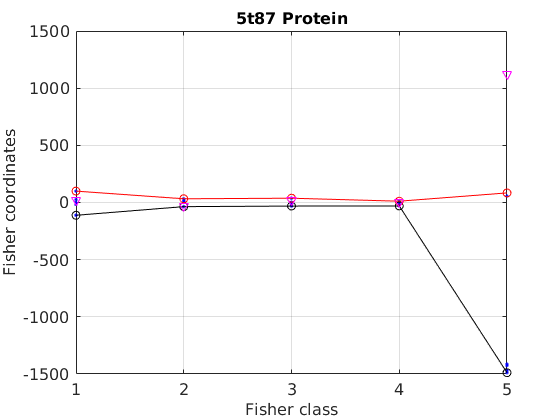

Supplement: Supplementary file 1 [file molecules-25-02467-s001.zip › Figure 6 - Supplementary Material/Figure 6 - S14 - 5t87.tif]

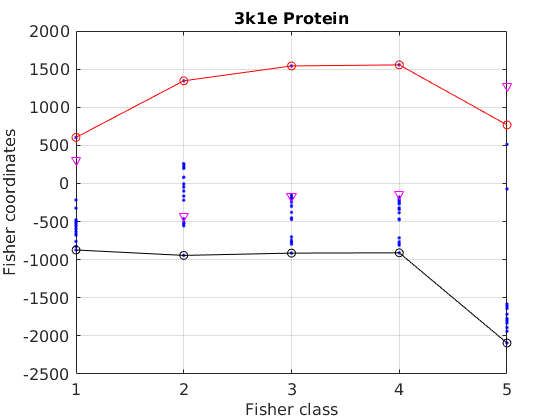

Supplement: Supplementary file 1 [file molecules-25-02467-s001.zip › Figure 6 - Supplementary Material/Figure 6 - S15 - 3k1e.tif]

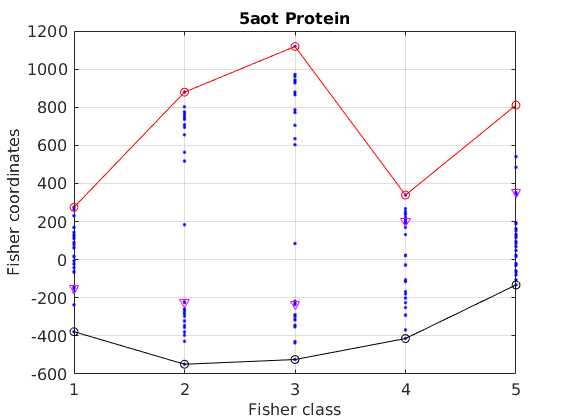

Supplement: Supplementary file 1 [file molecules-25-02467-s001.zip › Figure 6 - Supplementary Material/Figure 6 - S16 - 5aot.tif]

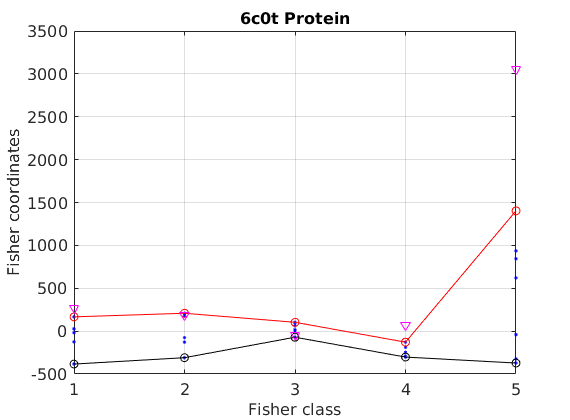

Supplement: Supplementary file 1 [file molecules-25-02467-s001.zip › Figure 6 - Supplementary Material/Figure 6 - S17 - 6c0t.tif]

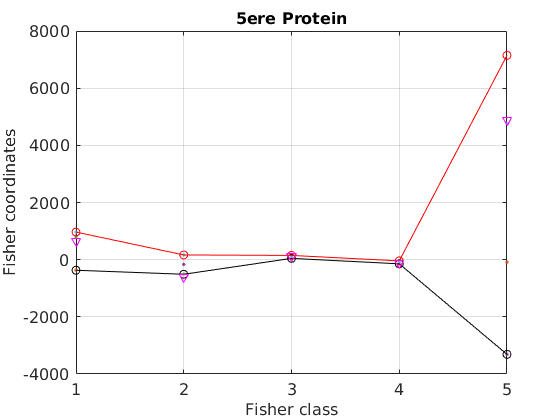

Supplement: Supplementary file 1 [file molecules-25-02467-s001.zip › Figure 6 - Supplementary Material/Figure 6 - S18 - 5ere.tif]

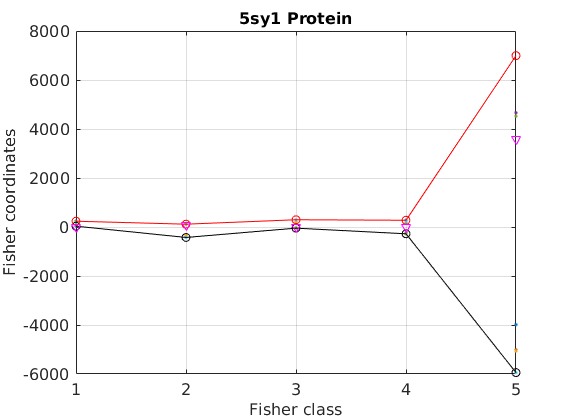

Supplement: Supplementary file 1 [file molecules-25-02467-s001.zip › Figure 6 - Supplementary Material/Figure 6 - S19 - 5sy1.tif]

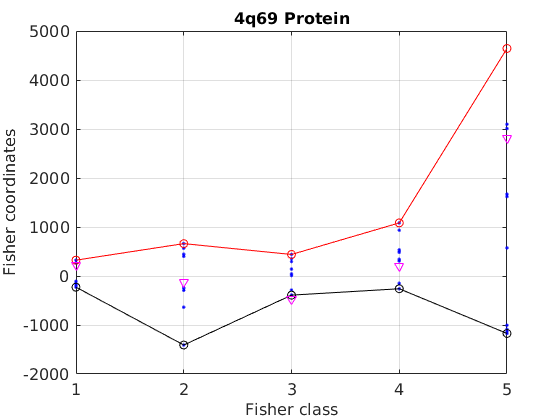

Supplement: Supplementary file 1 [file molecules-25-02467-s001.zip › Figure 6 - Supplementary Material/Figure 6 - S2 - 4q69.tif]

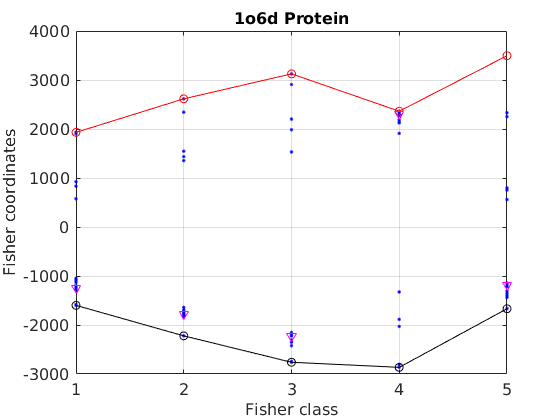

Supplement: Supplementary file 1 [file molecules-25-02467-s001.zip › Figure 6 - Supplementary Material/Figure 6 - S20 - 1o6d.tif]

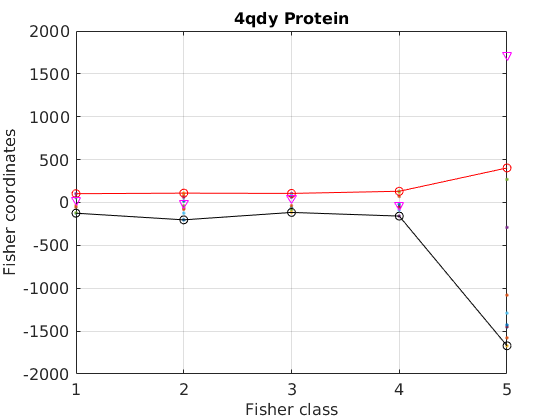

Supplement: Supplementary file 1 [file molecules-25-02467-s001.zip › Figure 6 - Supplementary Material/Figure 6 - S3 - 4qdy.tif]

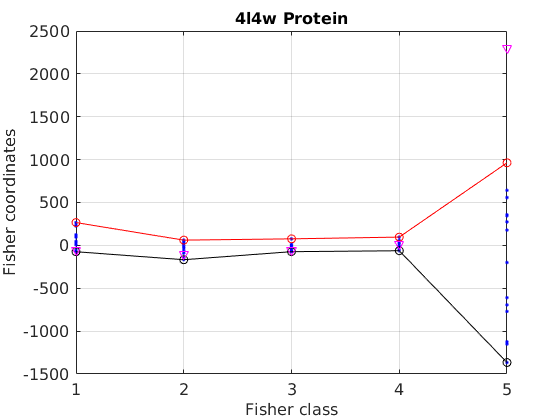

Supplement: Supplementary file 1 [file molecules-25-02467-s001.zip › Figure 6 - Supplementary Material/Figure 6 - S4 - 4l4w.tif]

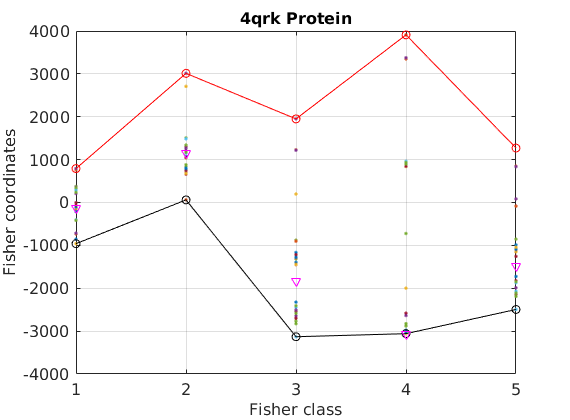

Supplement: Supplementary file 1 [file molecules-25-02467-s001.zip › Figure 6 - Supplementary Material/Figure 6 - S5 - 4qrk.tif]

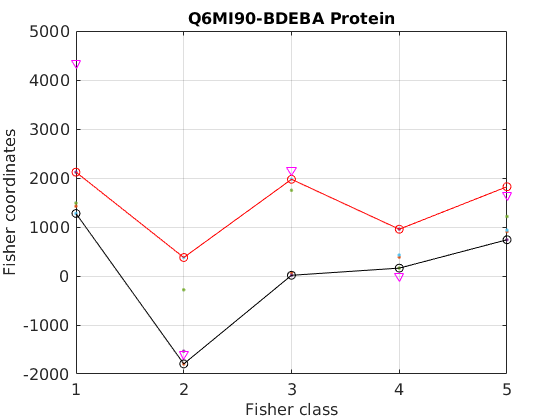

Supplement: Supplementary file 1 [file molecules-25-02467-s001.zip › Figure 6 - Supplementary Material/Figure 6 - S6 - Q6MI90_BDEBA.tif]

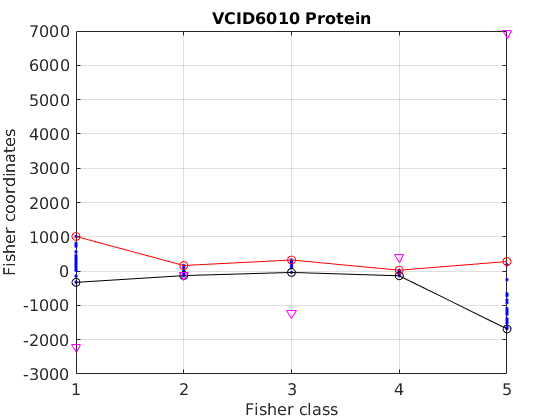

Supplement: Supplementary file 1 [file molecules-25-02467-s001.zip › Figure 6 - Supplementary Material/Figure 6 - S7 - VCID6010.tif]

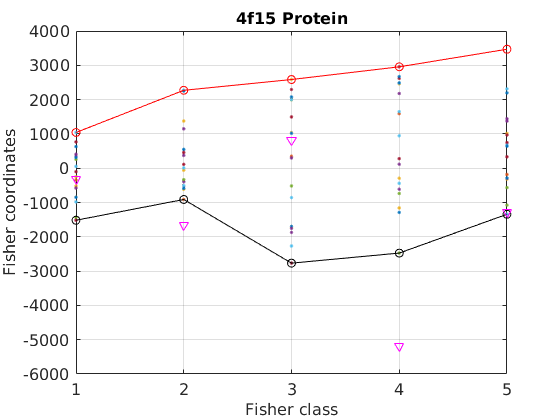

Supplement: Supplementary file 1 [file molecules-25-02467-s001.zip › Figure 6 - Supplementary Material/Figure 6 - S8 - 5f15.tif]

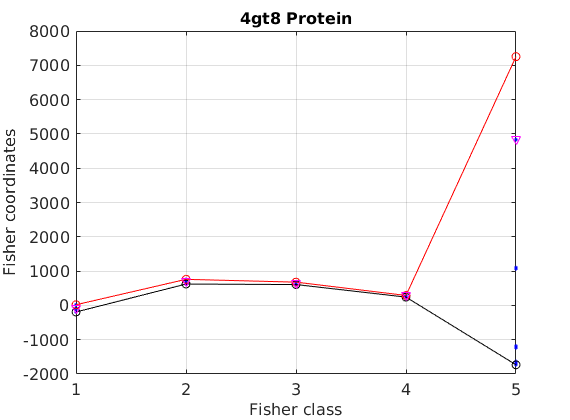

Supplement: Supplementary file 1 [file molecules-25-02467-s001.zip › Figure 6 - Supplementary Material/Figure 6 - S9 - 4gt8.tif]

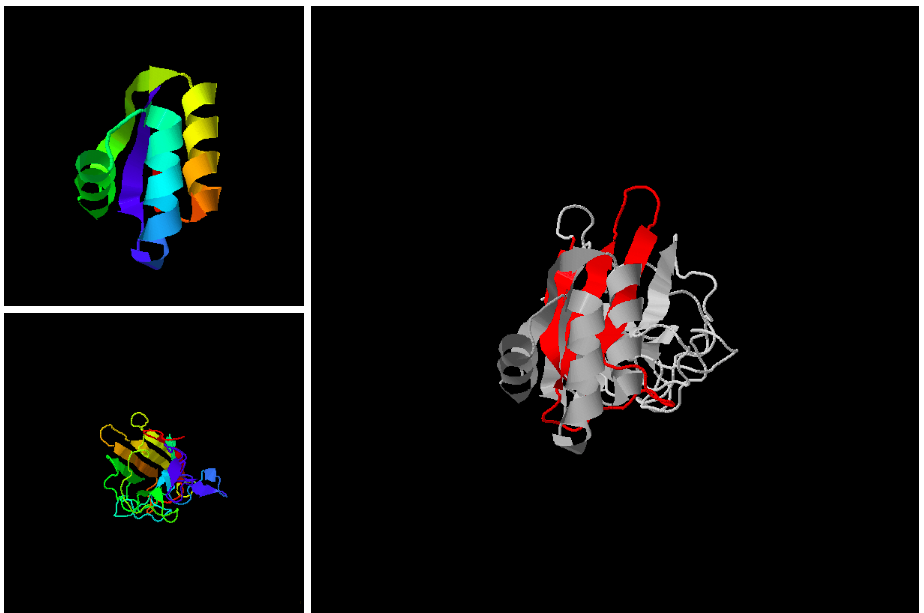

Supplement: Supplementary file 1 [file molecules-25-02467-s001.zip › Supplementary Figures/1ctf.tif]

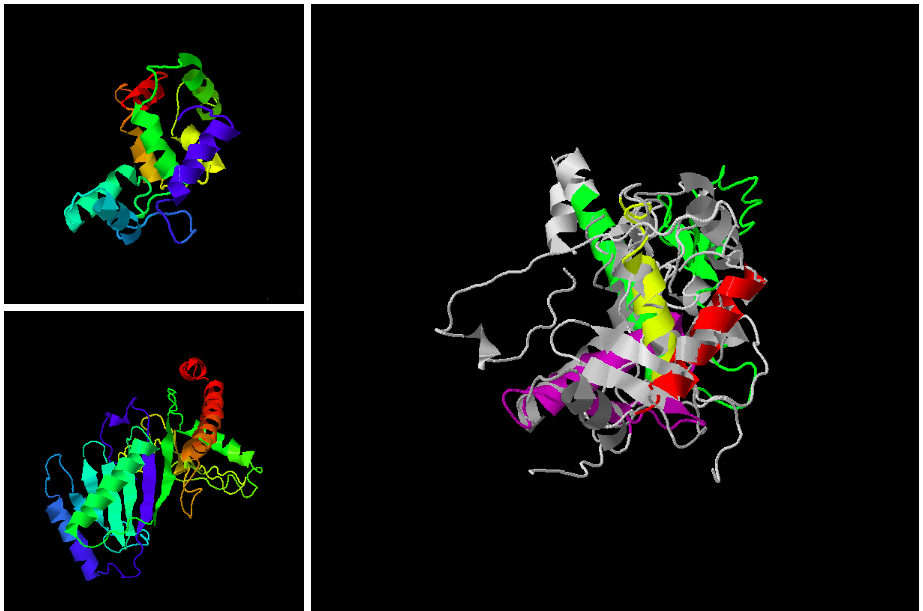

Supplement: Supplementary file 1 [file molecules-25-02467-s001.zip › Supplementary Figures/1o6d.tif]

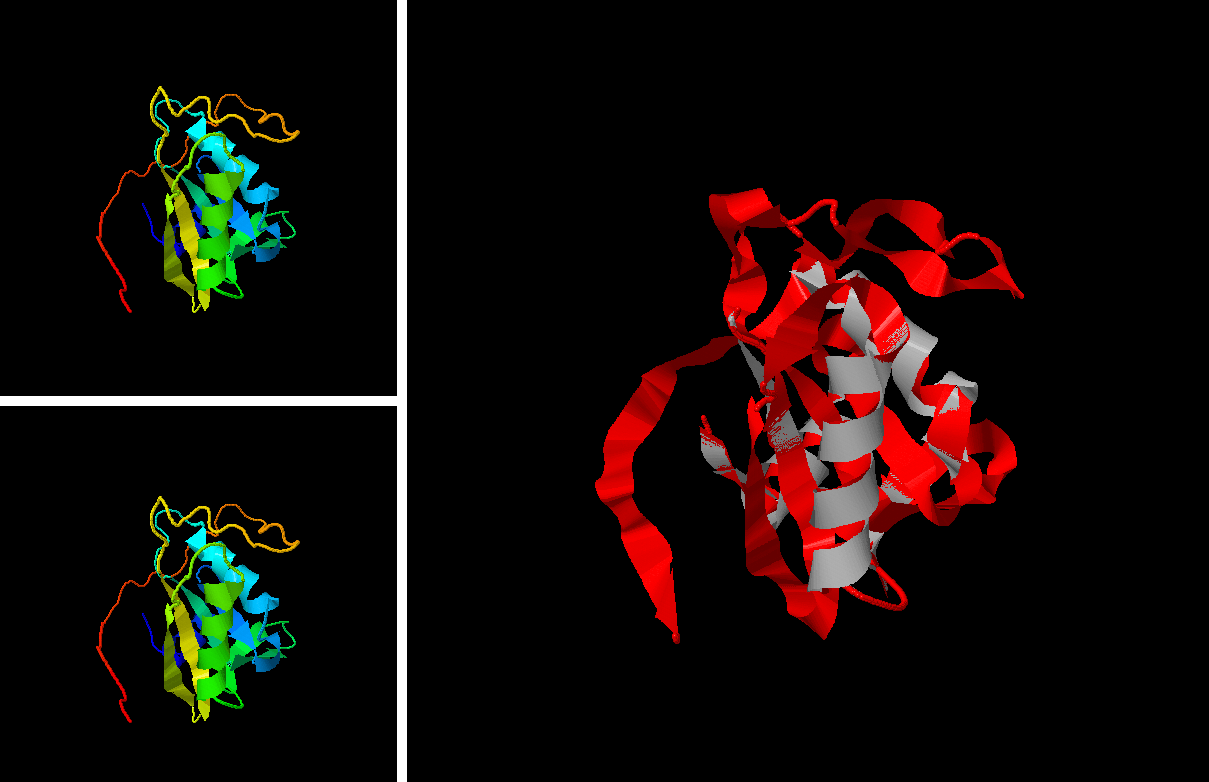

Supplement: Supplementary file 1 [file molecules-25-02467-s001.zip › Supplementary Figures/2kyy.tif]

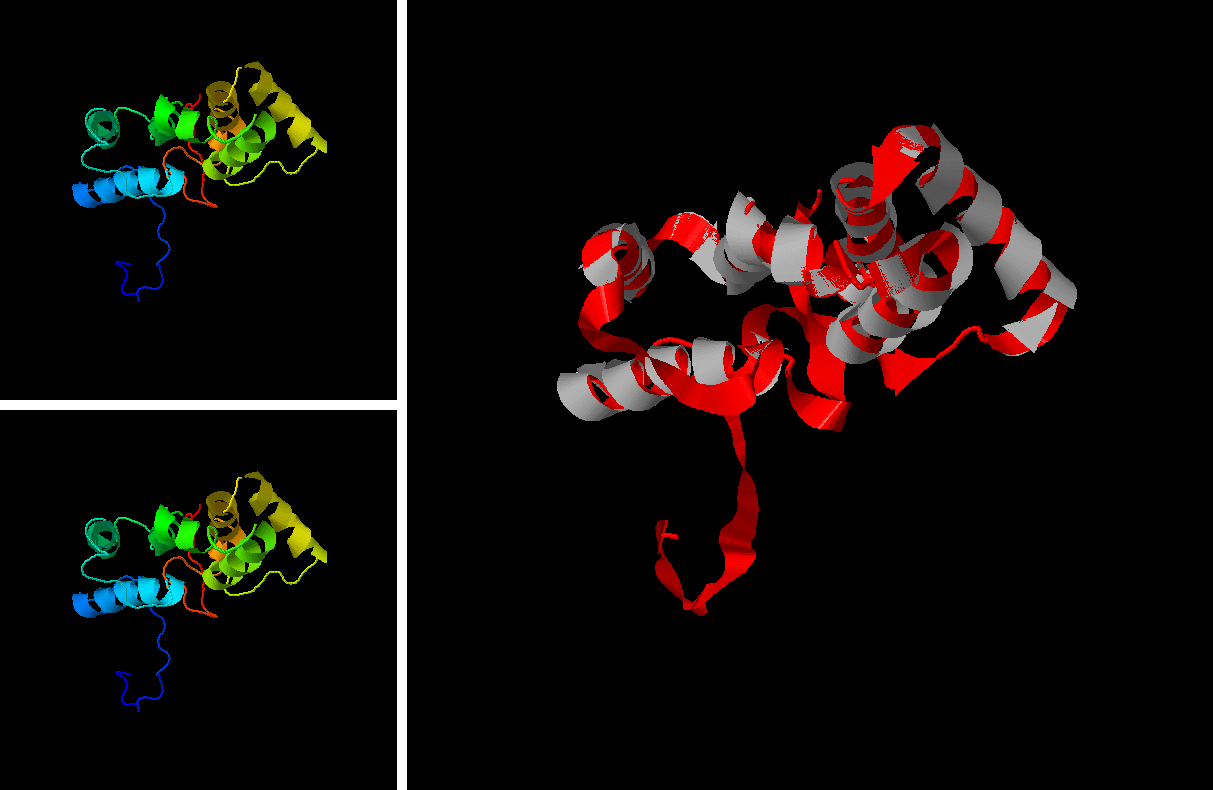

Supplement: Supplementary file 1 [file molecules-25-02467-s001.zip › Supplementary Figures/2l06.tif]

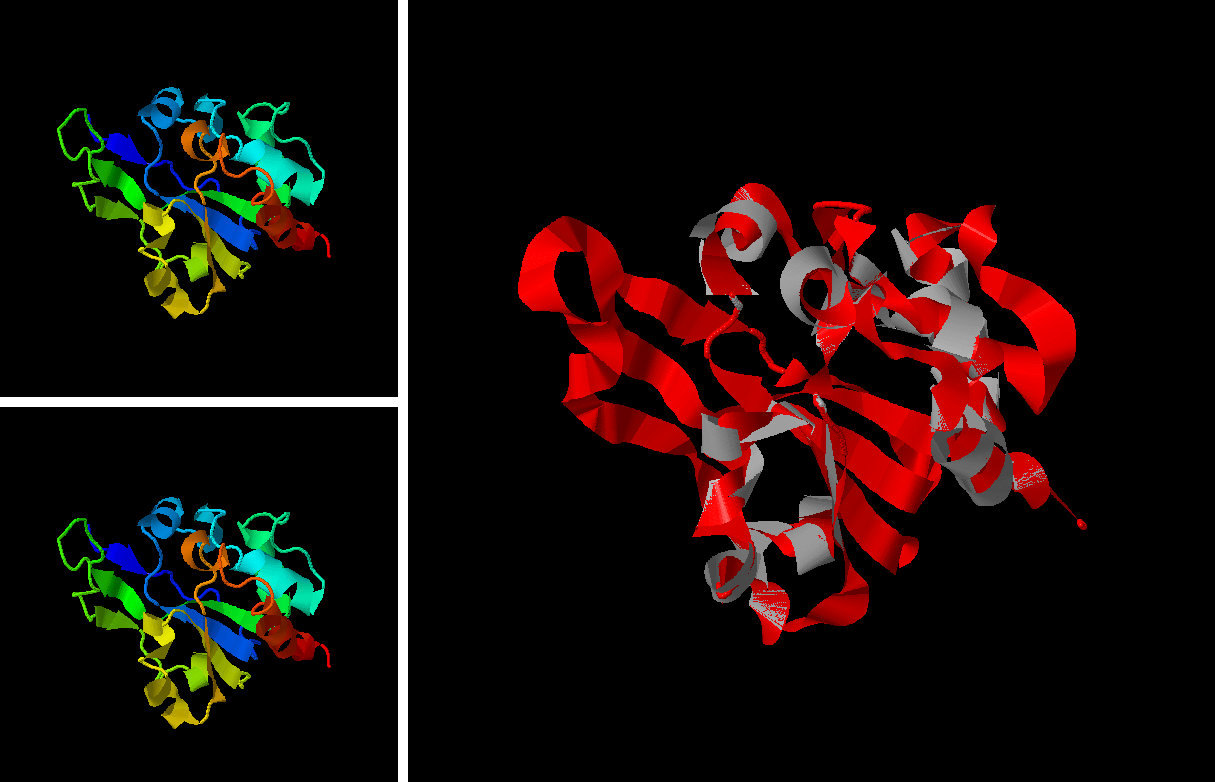

Supplement: Supplementary file 1 [file molecules-25-02467-s001.zip › Supplementary Figures/2l3f.tif]

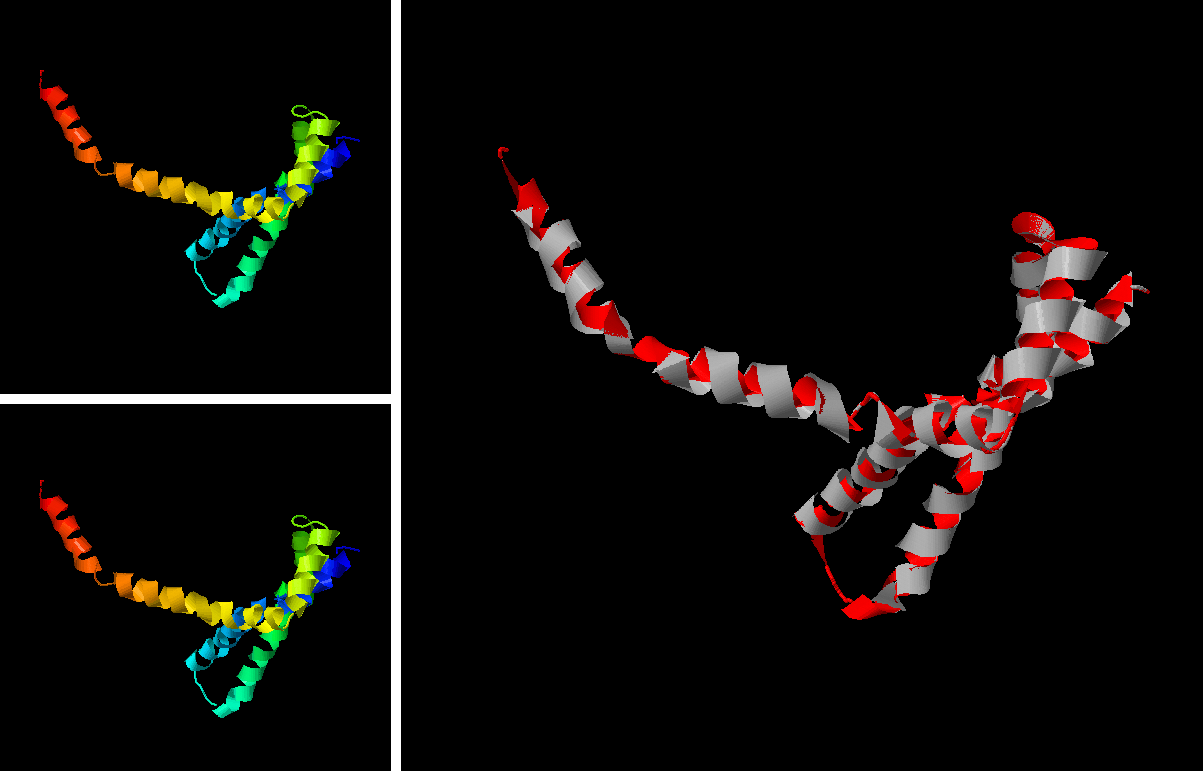

Supplement: Supplementary file 1 [file molecules-25-02467-s001.zip › Supplementary Figures/2x3o.tif]

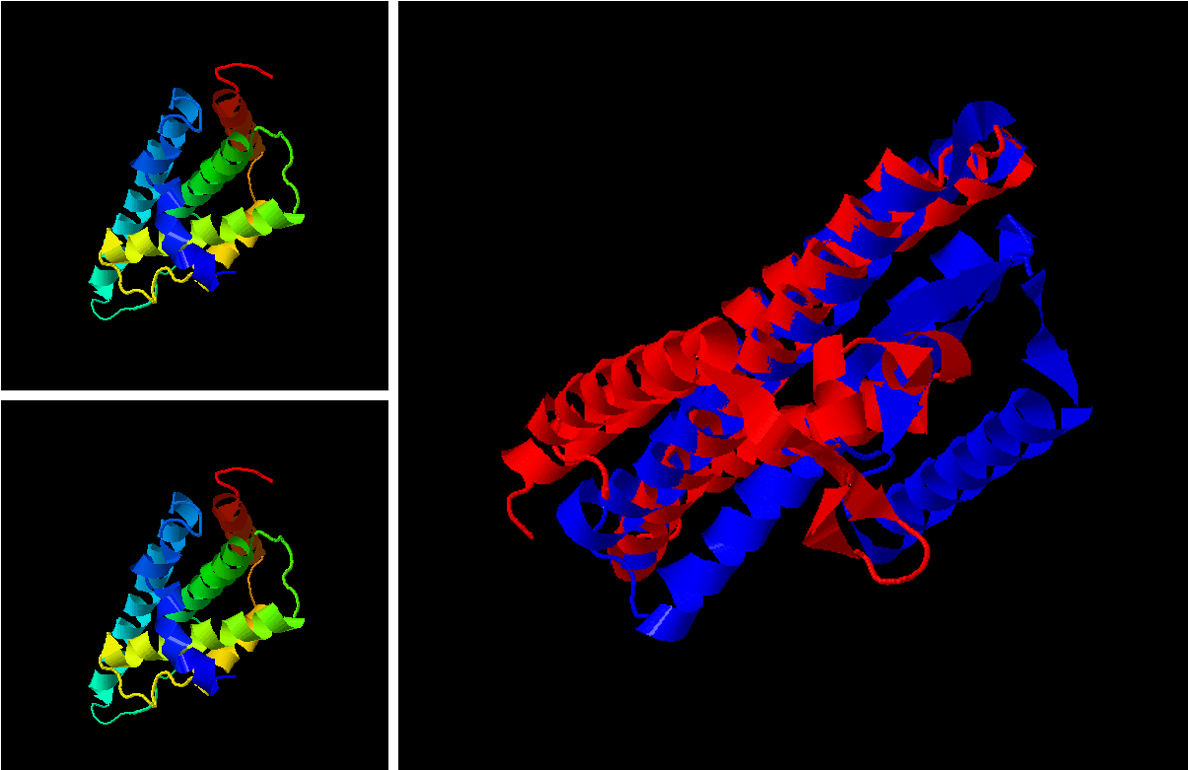

Supplement: Supplementary file 1 [file molecules-25-02467-s001.zip › Supplementary Figures/2xse.tif]

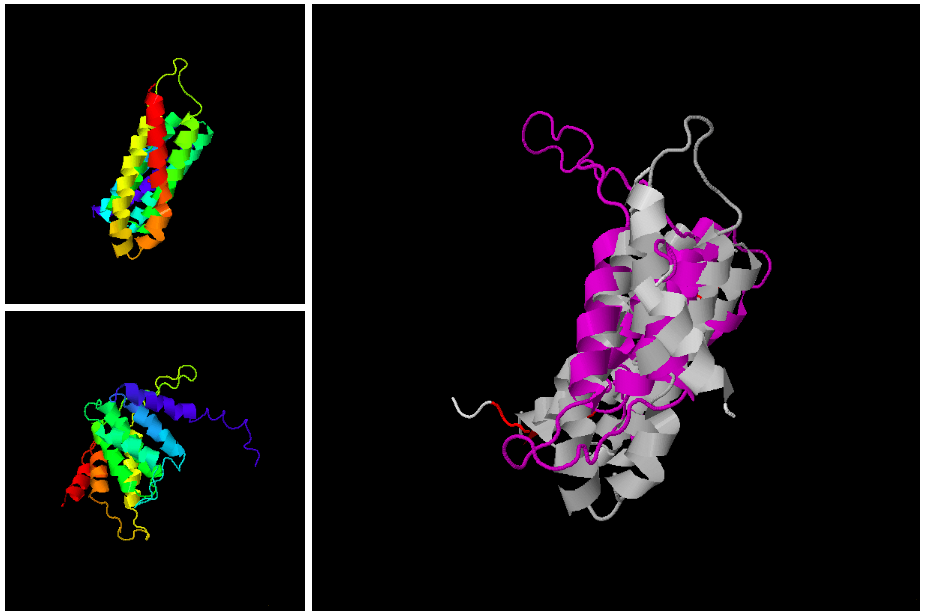

Supplement: Supplementary file 1 [file molecules-25-02467-s001.zip › Supplementary Figures/3k1e.tif]

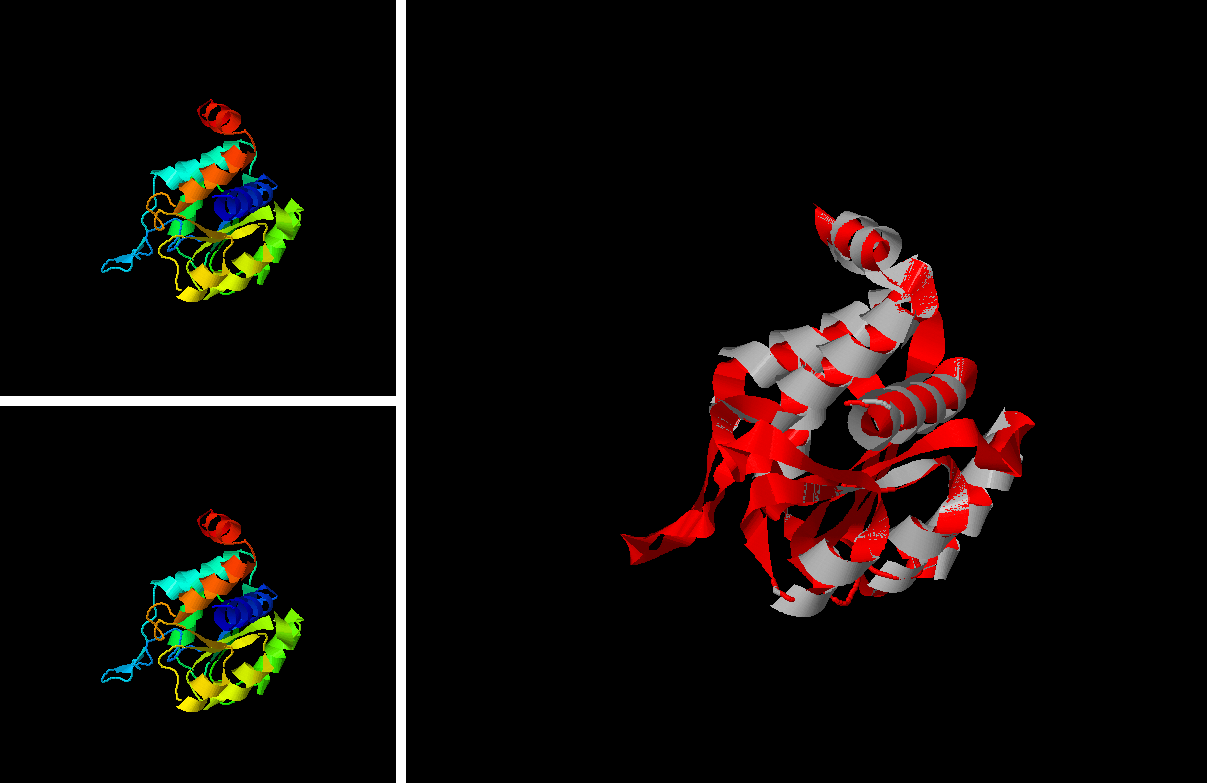

Supplement: Supplementary file 1 [file molecules-25-02467-s001.zip › Supplementary Figures/3n1u.tif]

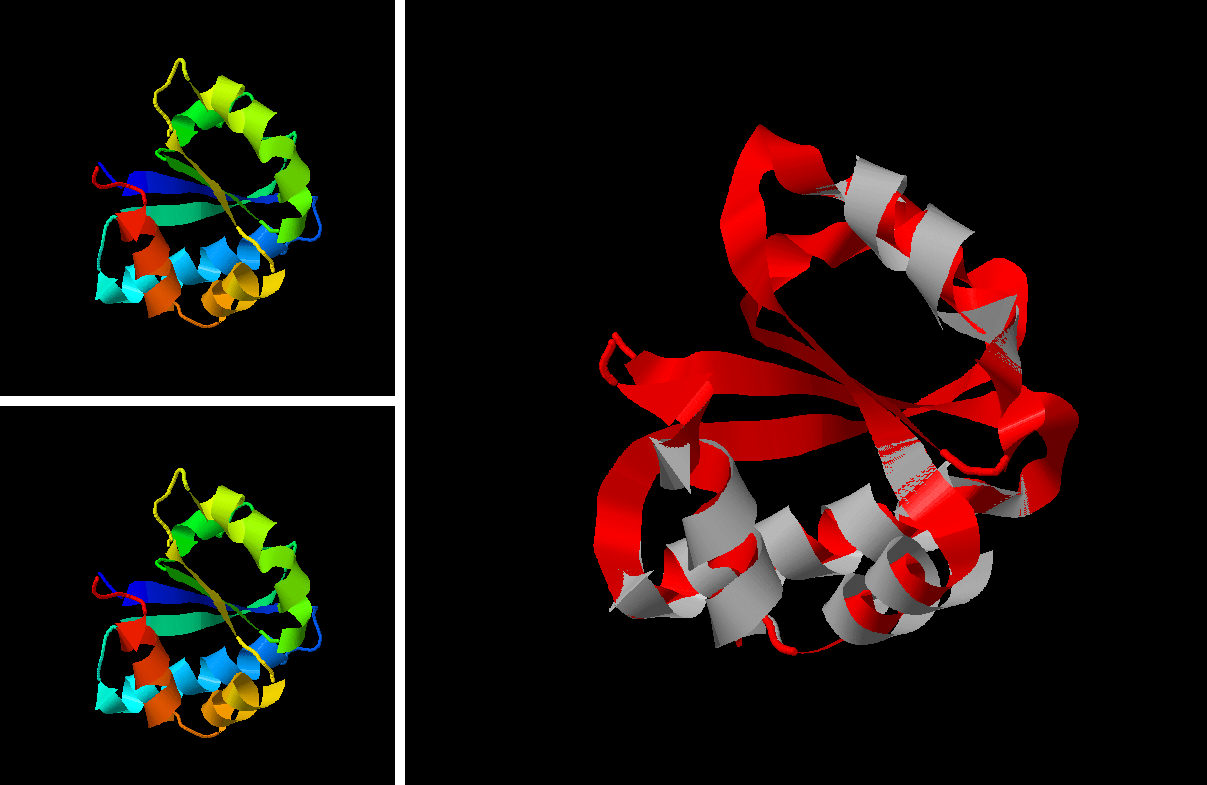

Supplement: Supplementary file 1 [file molecules-25-02467-s001.zip › Supplementary Figures/3nbm.tif]

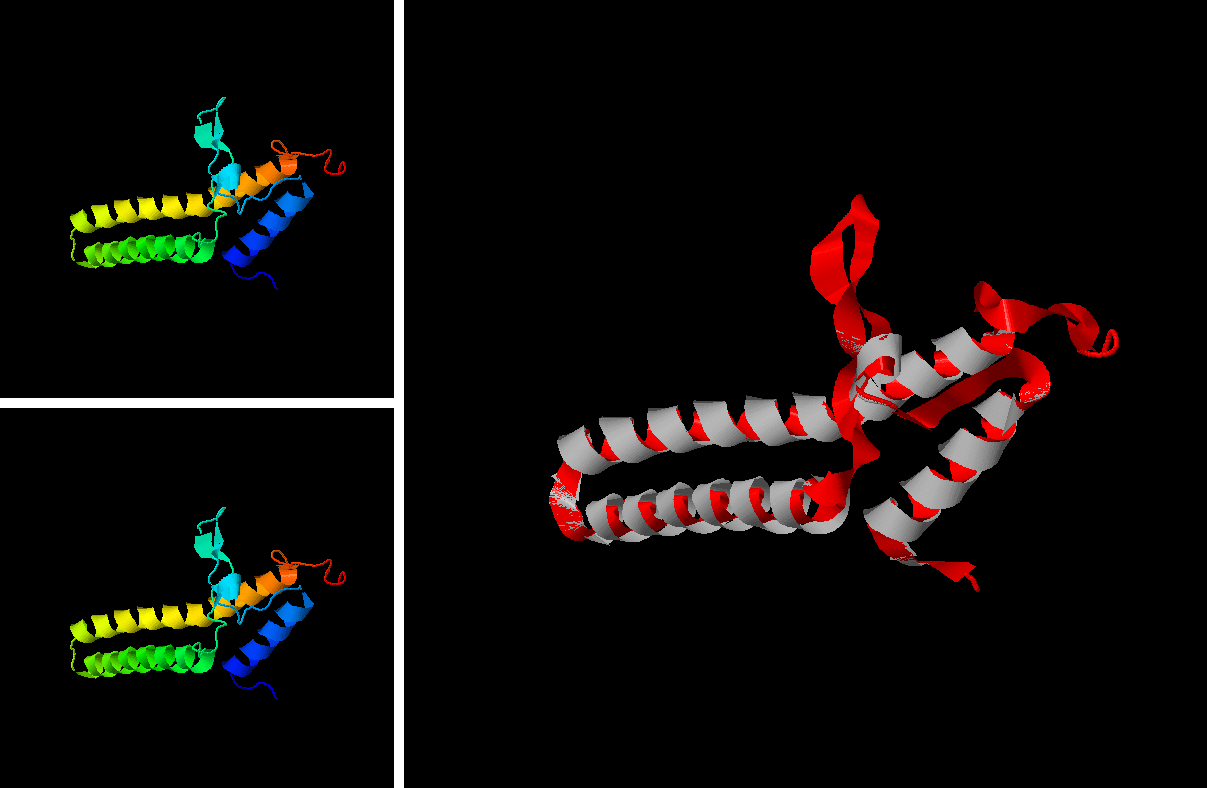

Supplement: Supplementary file 1 [file molecules-25-02467-s001.zip › Supplementary Figures/3nym.tif]

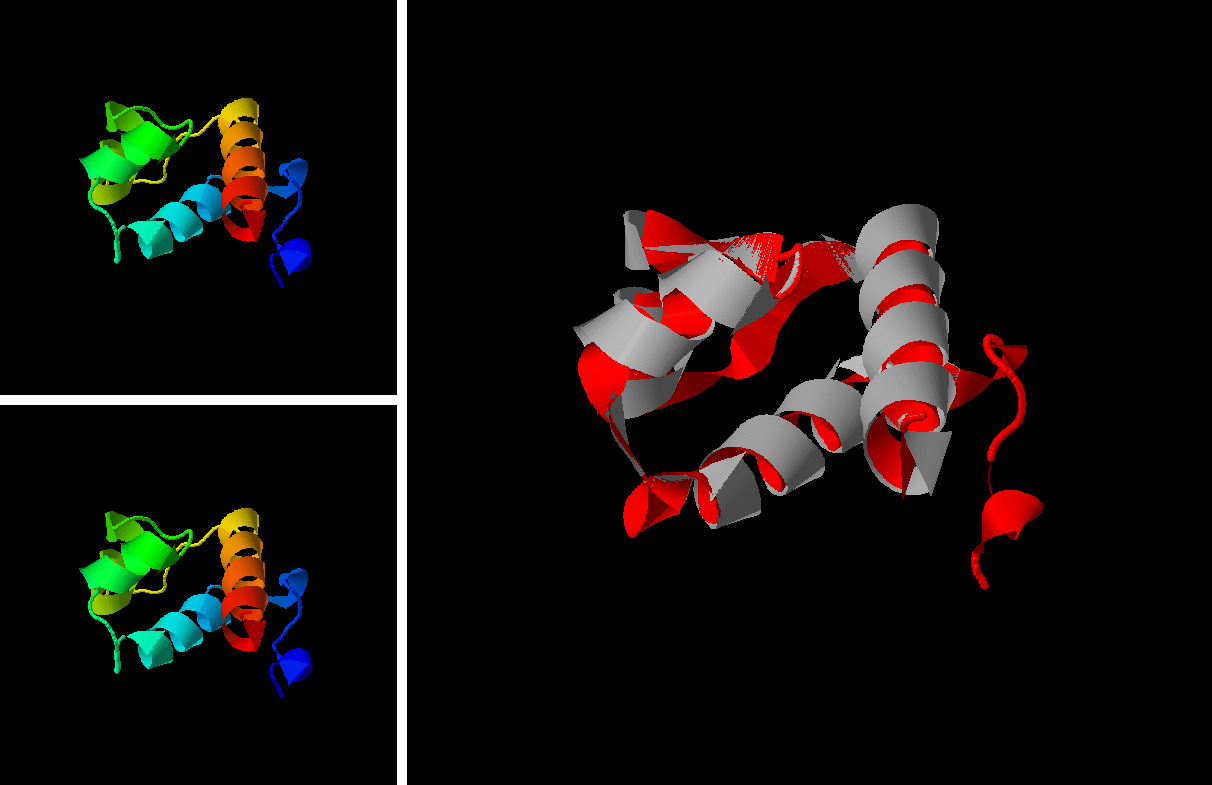

Supplement: Supplementary file 1 [file molecules-25-02467-s001.zip › Supplementary Figures/3nzl.tif]

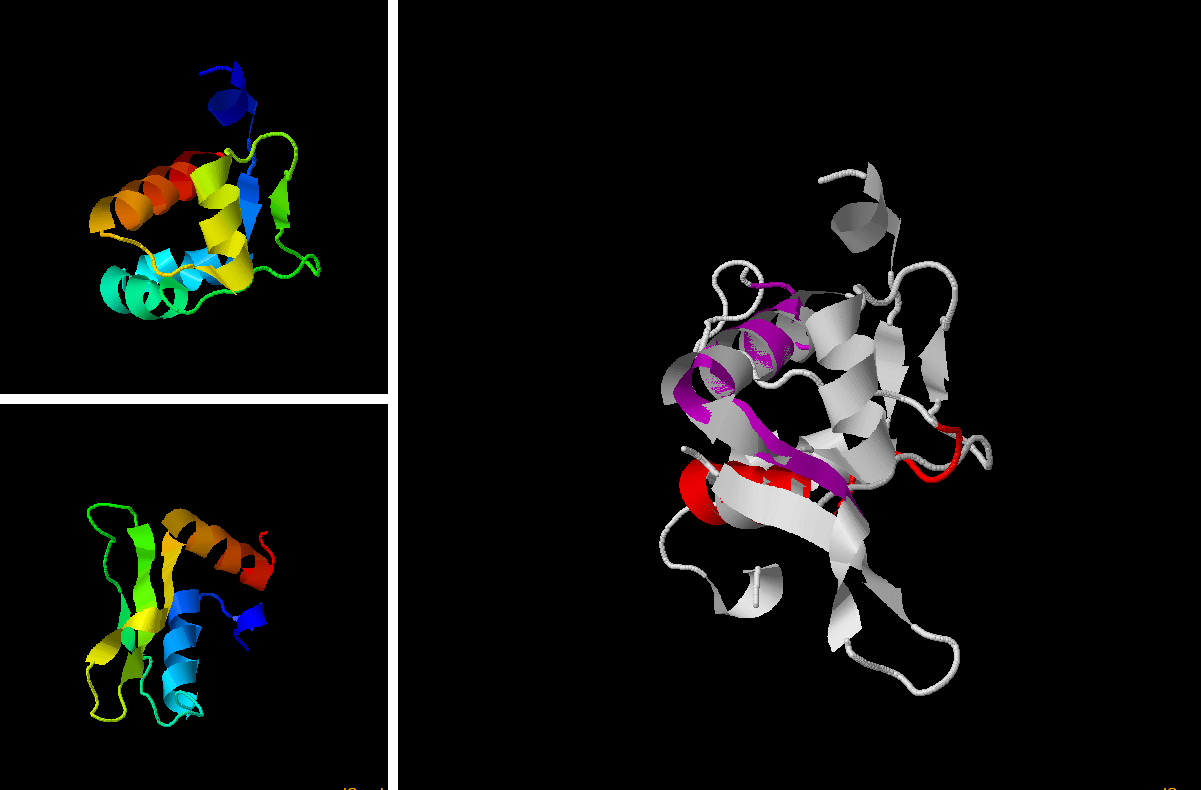

Supplement: Supplementary file 1 [file molecules-25-02467-s001.zip › Supplementary Figures/3obh.tif]

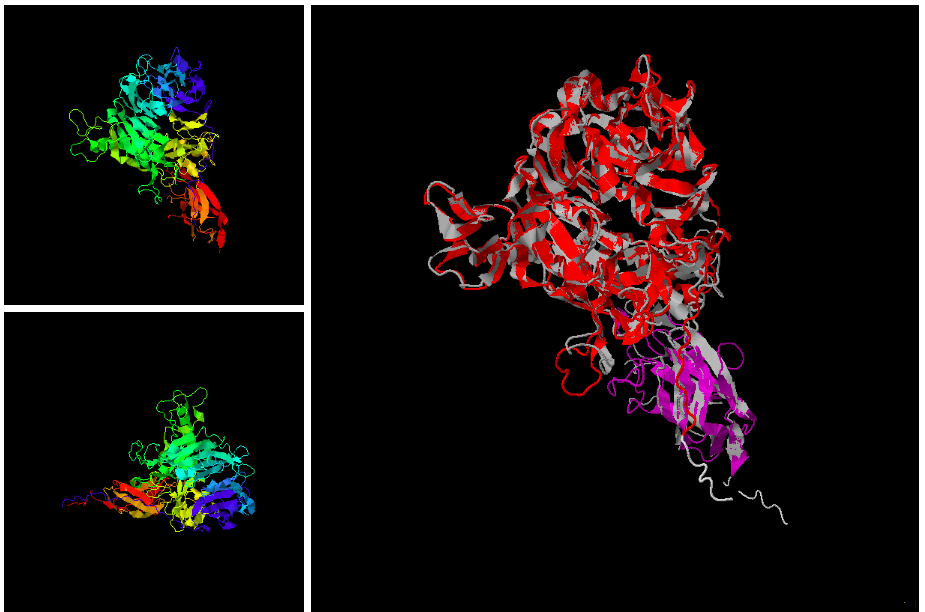

Supplement: Supplementary file 1 [file molecules-25-02467-s001.zip › Supplementary Figures/4gt8.tif]

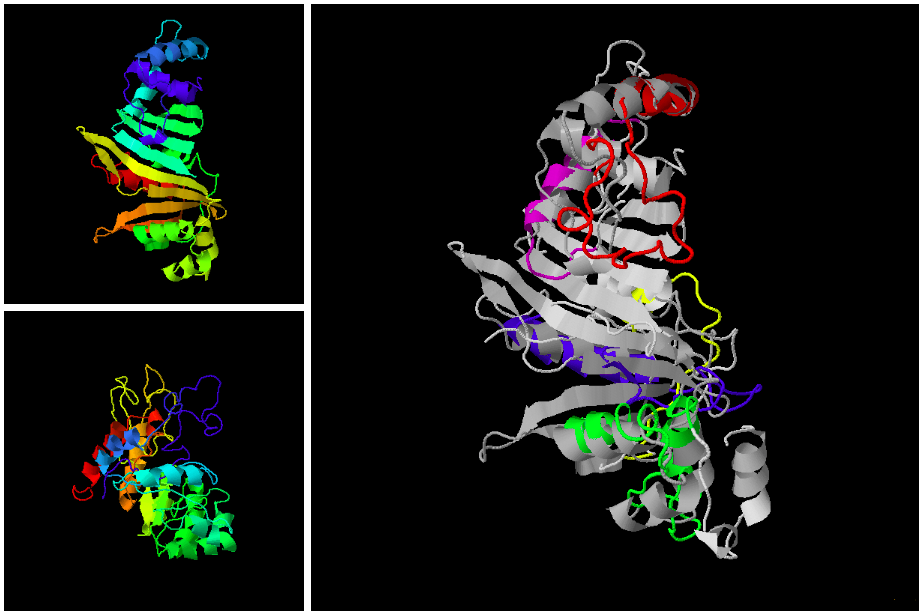

Supplement: Supplementary file 1 [file molecules-25-02467-s001.zip › Supplementary Figures/4l4w.tif]

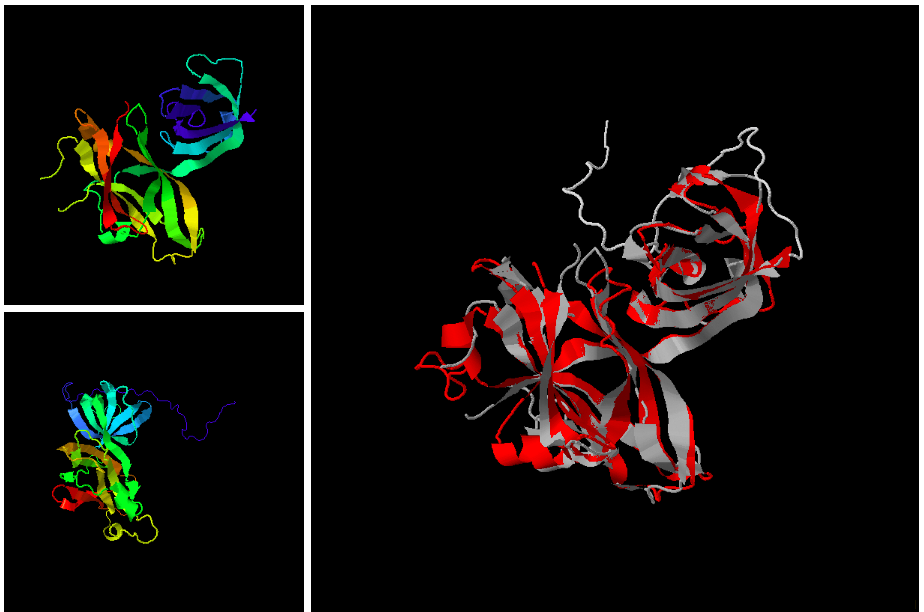

Supplement: Supplementary file 1 [file molecules-25-02467-s001.zip › Supplementary Figures/4pqx.tif]

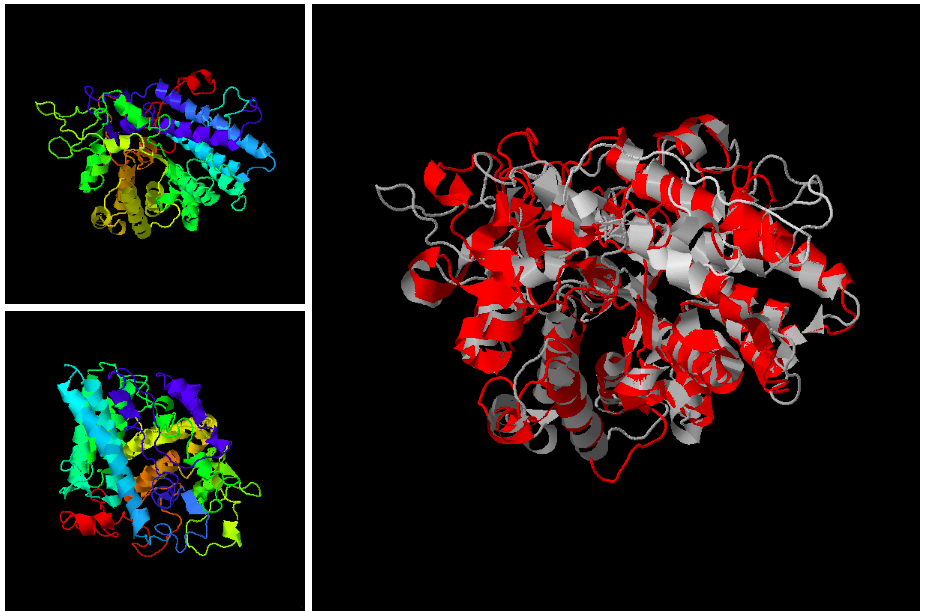

Supplement: Supplementary file 1 [file molecules-25-02467-s001.zip › Supplementary Figures/4q69.tif]

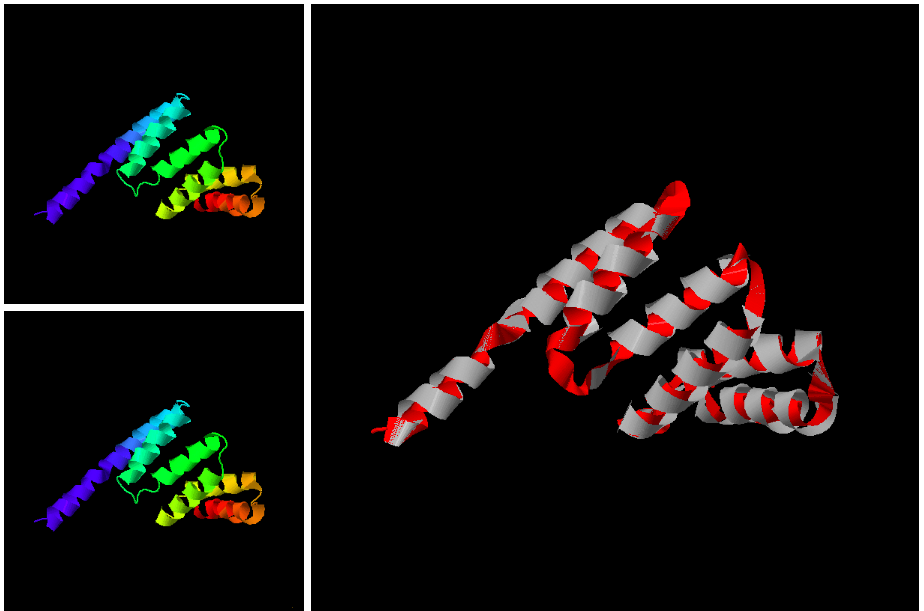

Supplement: Supplementary file 1 [file molecules-25-02467-s001.zip › Supplementary Figures/4qdy.tif]

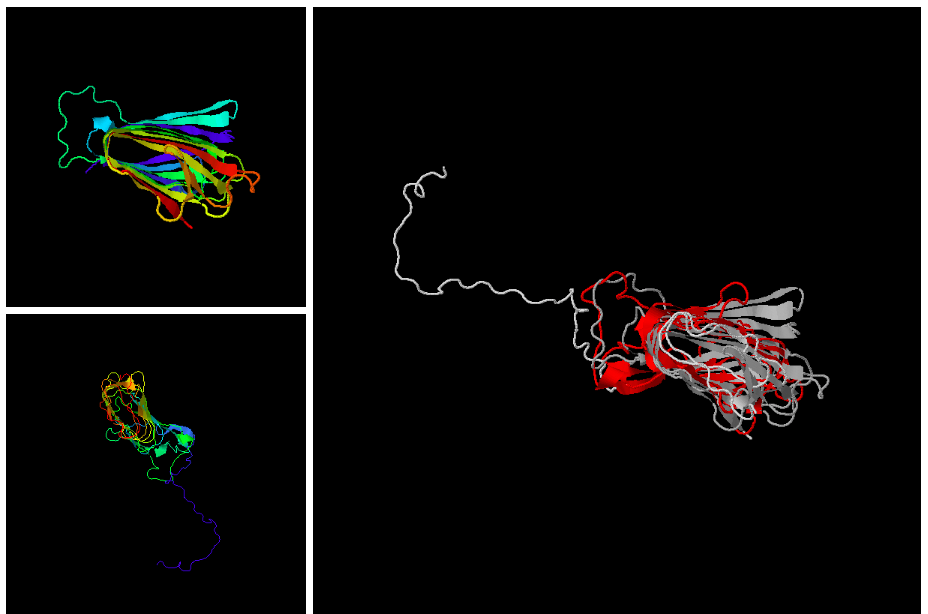

Supplement: Supplementary file 1 [file molecules-25-02467-s001.zip › Supplementary Figures/4qrk.tif]

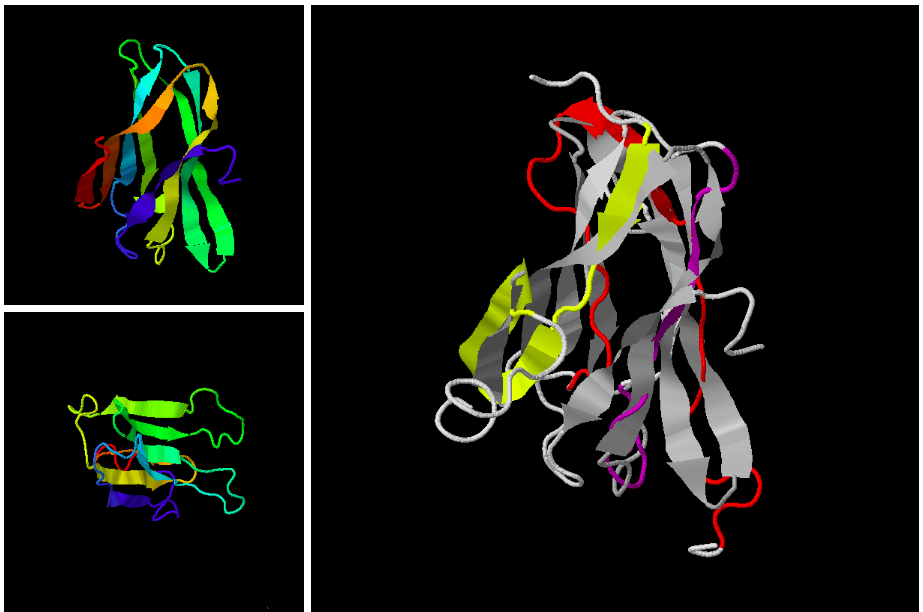

Supplement: Supplementary file 1 [file molecules-25-02467-s001.zip › Supplementary Figures/5aot.tif]

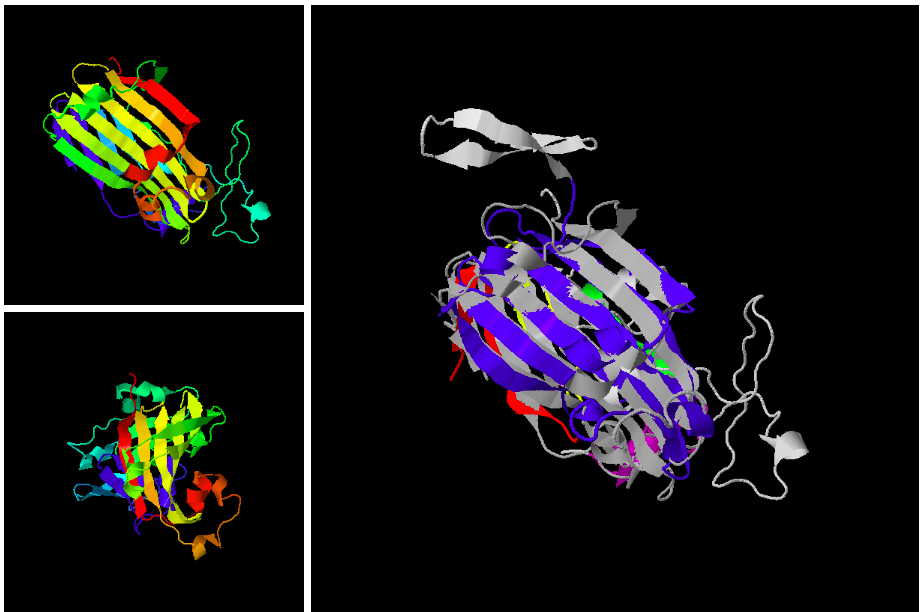

Supplement: Supplementary file 1 [file molecules-25-02467-s001.zip › Supplementary Figures/5dg9.tif]

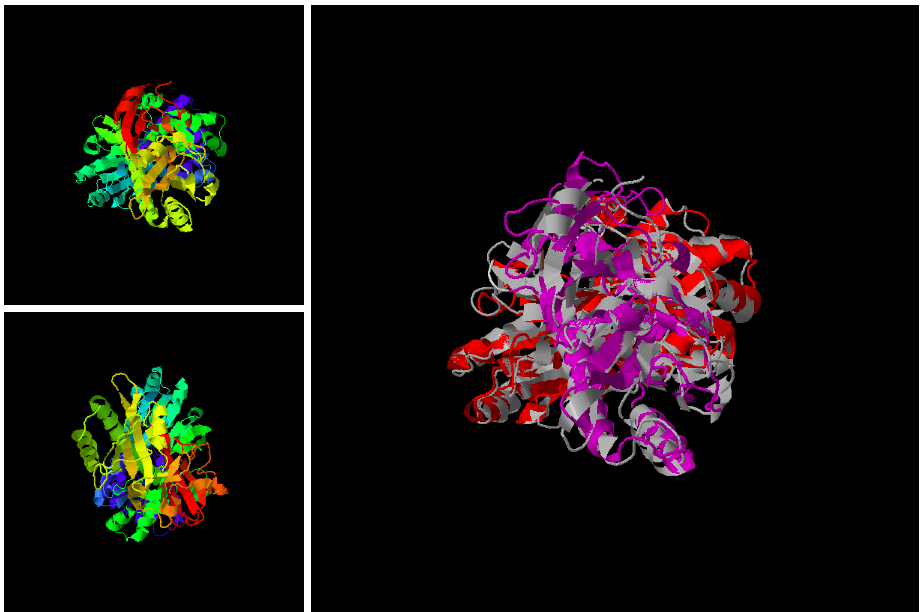

Supplement: Supplementary file 1 [file molecules-25-02467-s001.zip › Supplementary Figures/5ere.tif]

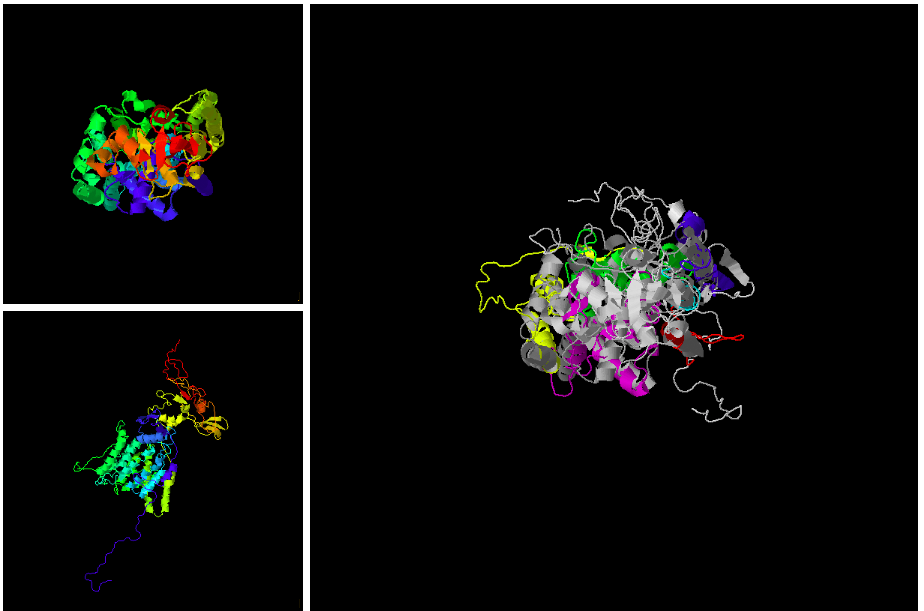

Supplement: Supplementary file 1 [file molecules-25-02467-s001.zip › Supplementary Figures/5fl5.tif]

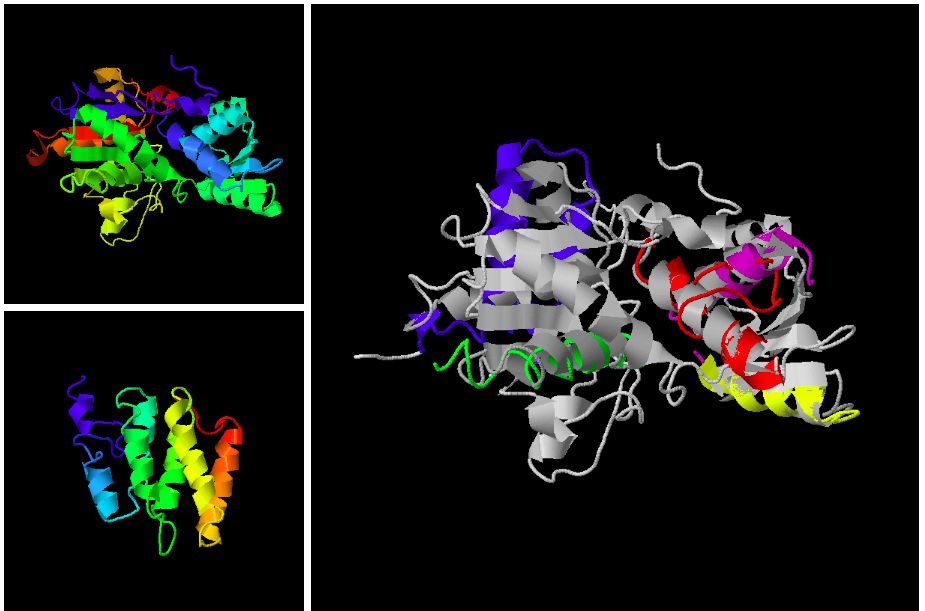

Supplement: Supplementary file 1 [file molecules-25-02467-s001.zip › Supplementary Figures/5j5v.tif]

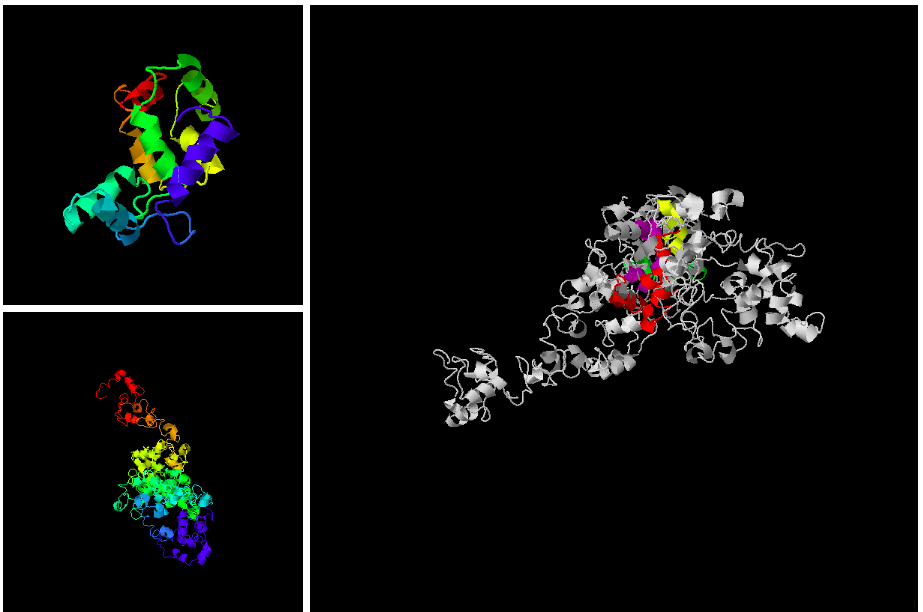

Supplement: Supplementary file 1 [file molecules-25-02467-s001.zip › Supplementary Figures/5sy1.tif]

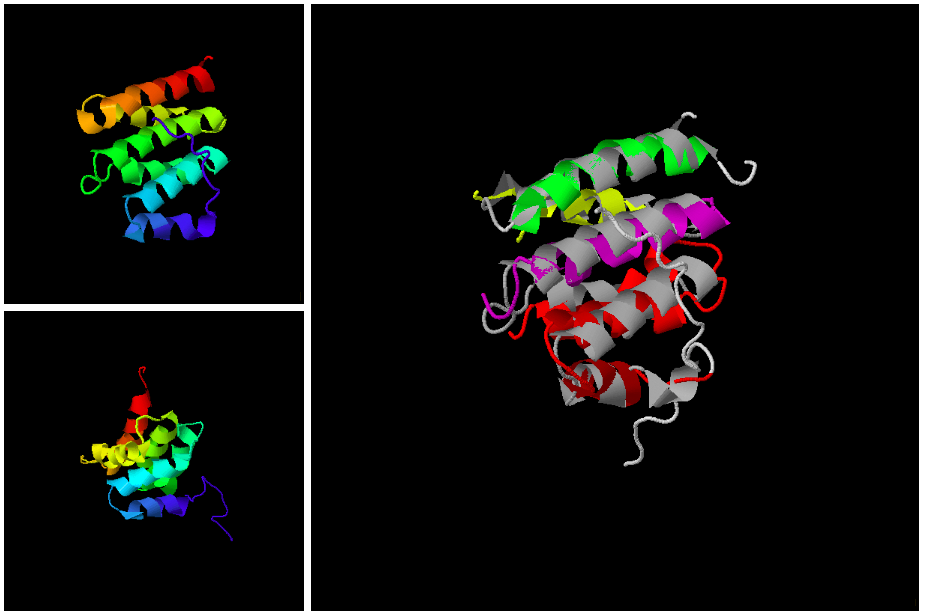

Supplement: Supplementary file 1 [file molecules-25-02467-s001.zip › Supplementary Figures/5t87.tif]

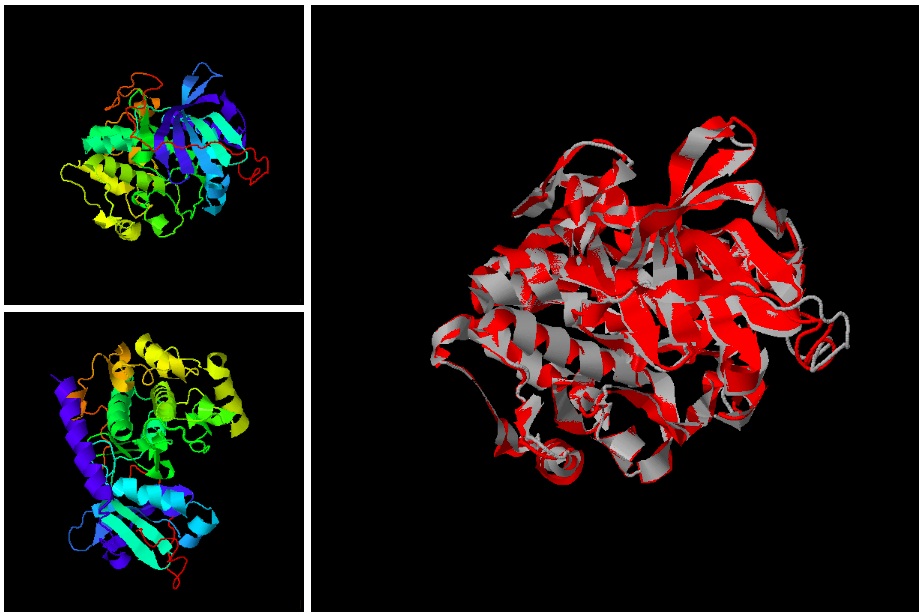

Supplement: Supplementary file 1 [file molecules-25-02467-s001.zip › Supplementary Figures/6c0t.tif]

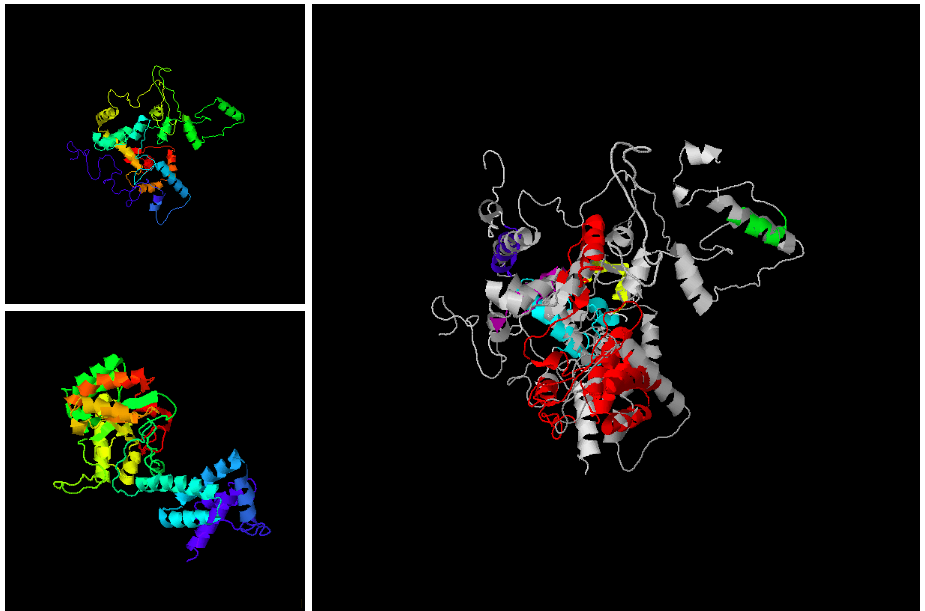

Supplement: Supplementary file 1 [file molecules-25-02467-s001.zip › Supplementary Figures/Q6MI90_BDEBA.tif]

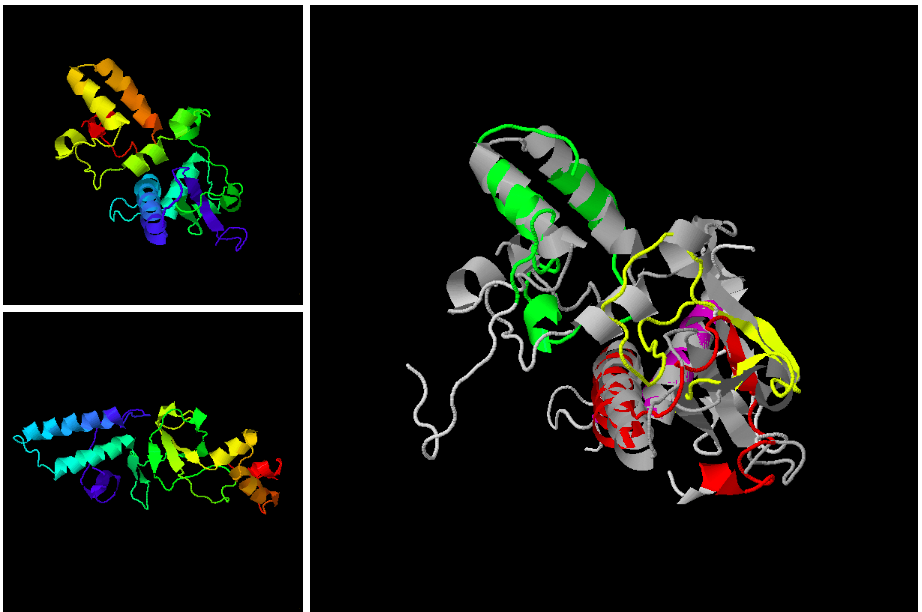

Supplement: Supplementary file 1 [file molecules-25-02467-s001.zip › Supplementary Figures/U1 Protein.tif]

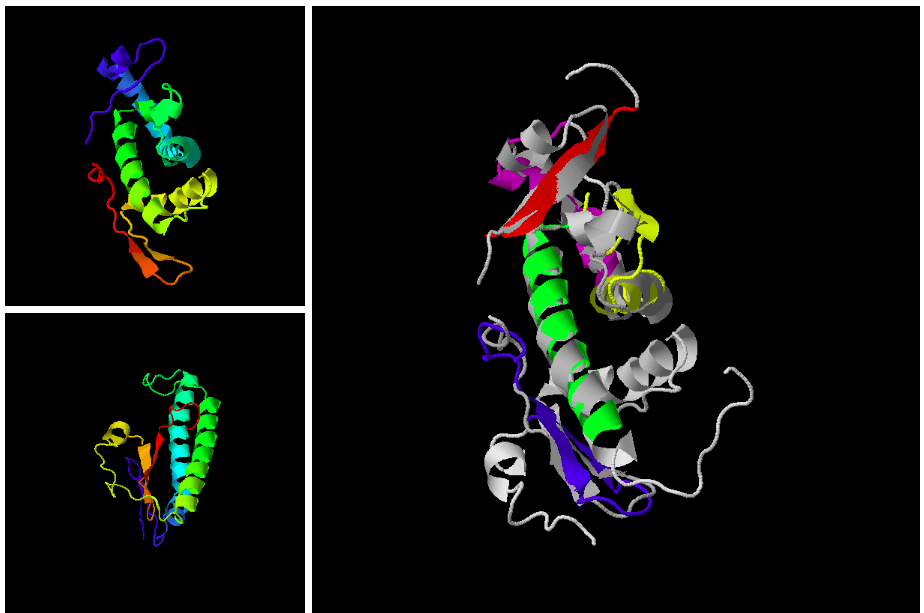

Supplement: Supplementary file 1 [file molecules-25-02467-s001.zip › Supplementary Figures/VCID6010.tif]
